# Caloric restriction remodels the hepatic chromatin landscape and bile acid metabolism by modulating the gut microbiota

**DOI:** 10.1186/s13059-023-02938-5

**Published:** 2023-04-30

**Authors:** Yun Fan, Hong Qian, Meijia Zhang, Chengzhe Tao, Zhi Li, Wenkai Yan, Yuna Huang, Yan Zhang, Qiaoqiao Xu, Xinru Wang, Paul A. Wade, Yankai Xia, Yufeng Qin, Chuncheng Lu

**Affiliations:** 1 State Key Laboratory of Reproductive Medicine, Center for Global Health, School of Public Health, Nanjing Medical University, Nanjing, 211166 China; 2 Key Laboratory of Modern Toxicology of Ministry of Education, School of Public Health, Nanjing Medical University, Nanjing, 211166 China; 3 Department of Microbes and Infection, School of Public Health, Nanjing Medical University, Nanjing, 211166 China; 4 Eukaryotic Transcriptional Regulation Group, Epigenetics and Stem Cell Biology Laboratory, National Institute of Environmental Health Sciences, Research Triangle Park, NC 27709 USA

**Keywords:** Caloric restriction, Gut microbiota, Hepatic epigenome, Bile acid metabolism, Metabolic adaptation

## Abstract

**Background:**

Caloric restriction (CR) has been known to promote health by reprogramming metabolism, yet little is known about how the epigenome and microbiome respond during metabolic adaptation to CR.

**Results:**

We investigate chromatin modifications, gene expression, as well as alterations in microbiota in a CR mouse model. Collectively, short-term CR leads to altered gut microbial diversity and bile acid metabolism, improving energy expenditure. CR remodels the hepatic enhancer landscape at genomic loci that are enriched for binding sites for signal-responsive transcription factors, including HNF4α. These alterations reflect a dramatic reprogramming of the liver transcriptional network, including genes involved in bile acid metabolism. Transferring CR gut microbiota into mice fed with an obesogenic diet recapitulates the features of CR-related bile acid metabolism along with attenuated fatty liver.

**Conclusions:**

These findings suggest that CR-induced microbiota shapes the hepatic epigenome followed by altered expression of genes responsible for bile acid metabolism.

**Supplementary Information:**

The online version contains supplementary material available at 10.1186/s13059-023-02938-5.

## Background

Nutrient content and caloric intake have been used to devise optimized diets to adapt for various phases of life. Calorie restriction (CR), intermittent fasting (IF), and time restricted feeding (TRF) are recognized as three main optimized diets in the treatment of metabolic disease. Forty percent CR nutritious diet improves metabolic adaptation and retards the onset of multiple age-associated diseases in diverse species [1]. A common characteristic of above metabolic improvements is mediated by the functional gut microbiota [2, 3]. The gut microbiota influences host metabolism through the alterations of metabolites, in part by mediating the gastrointestinal system as well as other distant organs [4, 5]. It generates numerous bioactive metabolites consisting of short-chain fatty acids (SCFAs), choline metabolites, vitamins, bile acids (BAs), which are dependent on the host nutrition, genome, and life-style [6, 7]. BA synthesis mainly functions via the classical pathway and the alternative pathway, initiated by cytochrome P450 cholesterol 7α-hydroxylase (CYP7A1) and 27α -hydroxylase (CYP27A1), respectively [8]. BAs alter host metabolism by binding to nuclear receptors to reprogram transcriptional signaling cascades regulating expression of genes responsible for bile acid, lipid, and carbohydrate metabolism as well as energy balance [9].

In addition, diet-microbiota interactions have been shown to alter the epigenome in multiple tissues including histone modifications [10]. Although epigenetic modifications are relatively stable and cell-type specific, these alterations still need to be flexibly fine-tuned in response to environmental challenges and can impact gene expressions [11]. The hepatic transcriptome is exquisitely responsive to nutritional signals and undergoes reprogramming to maintain metabolic homeostasis. Underlying how the hepatic epigenome answer CR may uncover new clues in ameliorating metabolic disorders.

In this study, we employed a mouse model under 40% CR nutritious diet to dig out molecular patterns of the interaction of host gut microbiota, epigenome, and BAs. Multiply improved effects of short-term CR on metabolic adaptations were explored. The composition of gut microbiota and its bioactive metabolites BAs were altered by CR. We applied ATAC-seq and ChIP-seq in combination with RNA-seq to investigate epigenomic and transcriptomic alteration during CR. Numerous loci with differential histone modification were identified, which were associated with gene expression that proved functionally relevant to BA metabolism. Moreover, transferring the bacteria from CR animals to diet-induced-obese mice is sufficient to recapitulate epigenetic changes and BA changes associated with hepatic adaptation and attenuated the fatty liver. Our study uncovers a mechanistic interplay between gut microbiota, host epigenome, gene expression, and BA synthesis in the modulation of metabolic adaptation in mice.

## Results

### Short-term caloric restriction improves metabolic adaptation

**Fig. 1:**
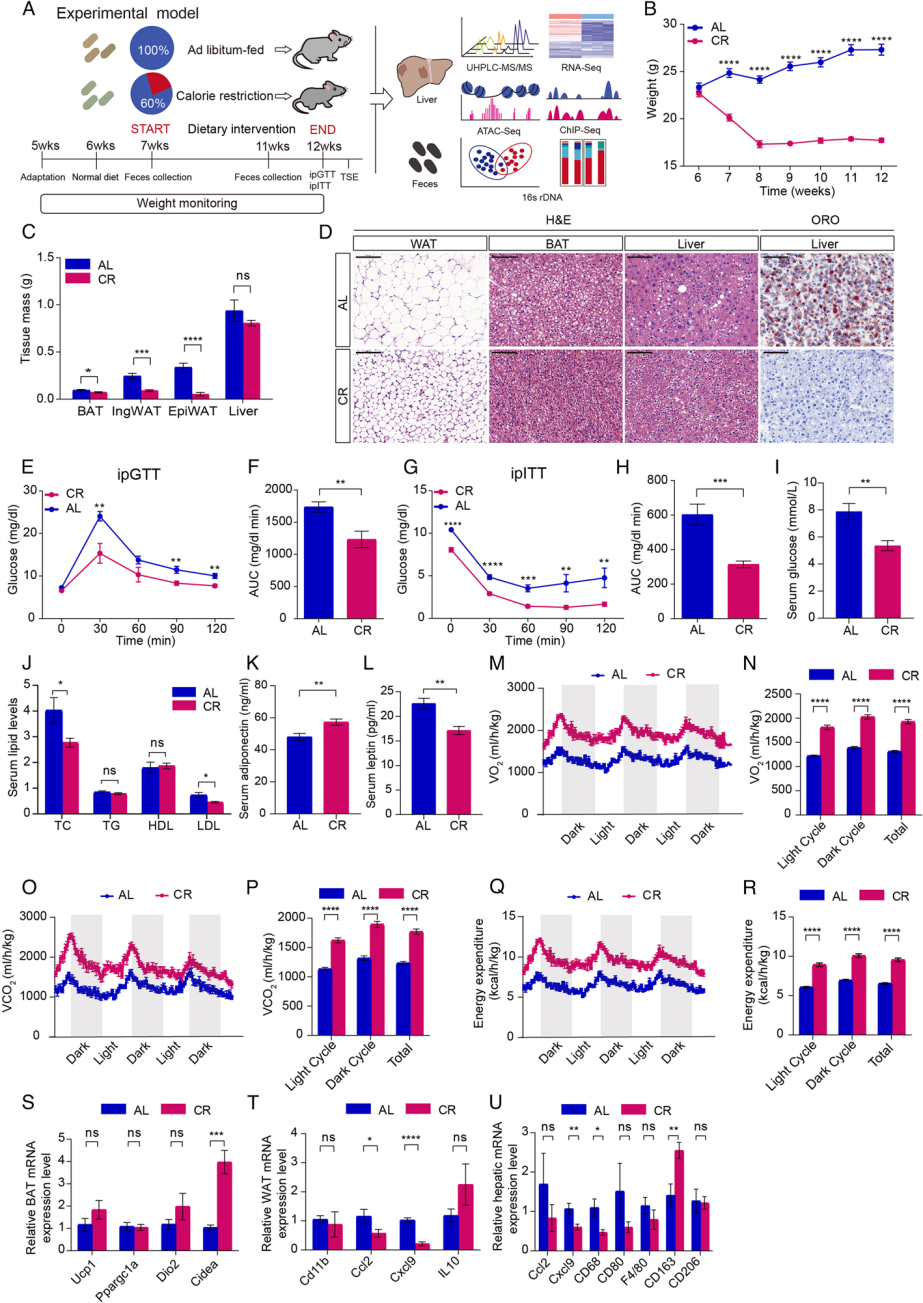
Short-term caloric restriction alters the metabolism parameters.** A** The pattern diagram of caloric restriction mouse model in the study. **B** Body weight of male mice in ad libitum (AL) and caloric restriction (CR) groups, respectively (*n* = 9–10 per group). **C** Brown adipose tissue (BAT), inguinal adipose tissue (IngWAT), epididymal adipose tissue (EpiWAT), and liver mass of male mice in AL and CR group (*n* = 9–10 per group). **D** Hematoxylin and eosin (H&E) staining of adipose tissue and liver in AL and CR groups (× 20 magnification; scale bar indicates 100 μm). BAT: Brown adipose tissue, WAT: White adipose tissue; Oil Red O staining of liver in AL and CR groups (× 20 magnification; scale bar indicates 100 μm). **E,F** IPGTT tests in male mice fed the AL or CR diets at 12 week and quantification of the AUC (*n* = 9–10 per group). **G–H** IPITT tests in male mice fed the AL or CR diets at 12 weeks and quantification of the AUC (*n* = 9–10 per group). **I** Serum glucose levels of male mice from AL or CR group (*n* = 8 per group). **J** Serum lipid levels of male mice from AL or CR group (*n* = 9–10 per group). TC: total cholesterol, TG: triglyceride, LDL: low-density lipoprotein, HDL: How-density lipoprotein. **K,L** Serum adiponectin and leptin levels of male mice from AL or CR group (*n* = 8 per group). **M–R** TSE phenoMaster cage analysis of oxygen consumption rate (VO_2_), carbon dioxide production (VCO_2_), and energy expenditure (EE) in male mice fed the AL or CR diets (*n* = 6 per group). **S–U** Relative mRNA expression levels of markers of adipose browning in BAT, and pro-inflammatory markers or anti-inflammatory markers in in WAT or liver. Significance was calculated using non-paired two-tailed Student’s *t* test. ∗ *p* < 0.05, ∗  ∗ *p* < 0.01, ∗  ∗  ∗ *p* < 0.001, ∗  ∗  ∗  ∗ *p* < 0.0001. Multiple testing correction was calculated. * *FDR* < 0.1, ** *FDR* < 0.05, *** *FDR* < 0.01, and **** *FDR* < 0.001 were determined statistically significant

To investigate the adaptive metabolic changes of CR, we fed mice with CR diet (40% less energy from carbohydrate) for 6 weeks (Fig. 1A). The energy density of the food is isoenergetic and the average daily energy intake of the CR group was calculated by the daily food intake × 0.6 (the energy density of the food in the control). Using this strategy, the mice in CR group will be 40% caloric restriction without affecting protein and fat consumption (Additional file 1: Table S1). Compared with the ad libitum (AL) group, CR significantly reduced body weight after 1 week and did not cause obvious malnutrition during the study (Fig. 1B). BAT mass and WAT mass (epididymal and inguinal WAT) significantly decreased in CR group, while liver mass decreased with no significant difference (Fig. 1C). Oil red O and H&E staining demonstrated that a decrease in lipid deposits in the CR mice liver sections (Fig. 1D). Consistent with previous studies [3, 12, 13], CR showed improved glucose-insulin homeostasis (Fig. 1E–H) along with lower serum levels of glucose (Fig. 1I) and total cholesterol (TC), low-density lipoprotein (LDL) (Fig. 1J). This glucose-insulin homeostasis is partly attributed to the inhibition of hepatic glucose capacity and the elevation of glucose consumption in the fat mass. Serum levels of the anti-inflammatory adipokine adiponectin were observably elevated in CR group (Fig. 1K). Additionally, lower serum fat-responsive hormone leptin was detected, suggesting decreased fat accumulation in the CR group (Fig. 1L). Using TSE phenoMaster cages, we measured oxygen consumption (VO_2_), carbon dioxide production (VCO_2_), and energy expenditure (EE). CR significantly increased VO_2_ (Fig. 1M,N) and VCO_2_ compared to the AL group (Fig. 1O,P). Higher EE indicated that more energy was released as heat in CR mice (Fig. 1Q,R). Real-time PCR confirmed that markers of adipose browning were increased in CR male mice (Fig. 1S), whereas pro-inflammatory markers were reduced and anti-inflammatory markers were increased in WAT or liver (Fig. 1T,U).

### Short-term caloric restriction manipulates the gut microbiota composition

**Fig. 2:**
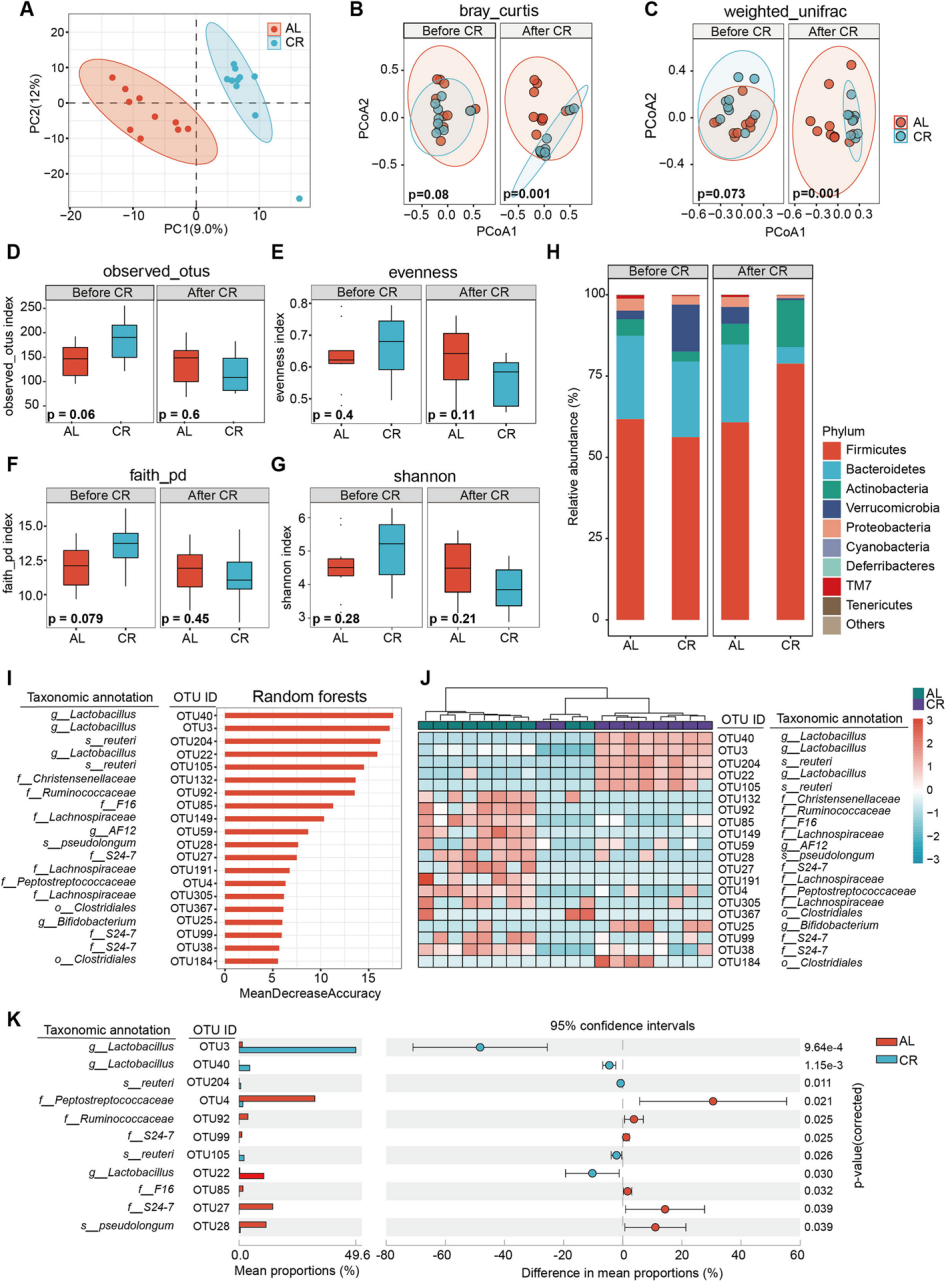
Short-term caloric restriction changes the gut microbiota composition.** A** Pairwise orthogonal projection to latent structure-discriminant analysis (OPLS-DA) analysis of fecal samples (*n* = 9–10 per group). **B,C** Principal coordinate analysis (PCoA) of the Bray–Curtis and weighted_unifrac distances of feces from AL or CR mice at 7 weeks and 11 weeks (*n* = 9–10 per group). **D–G** Indexes of observed_otus, evenness, faith_pd, Shannon of feces from AL or CR mice at 7 weeks and 11 weeks (*n* = 9–10 per group). **H** Comparison of phylum-level proportional abundance of feces from AL and CR mice at 7 weeks and 11 weeks (*n* = 9–10 per group). **I** Classification model between the AL and CR groups at 11 weeks based on random forests. **J** Heatmap of 20 OTUs which were highly predictive for the differentiation of the gut microbiota between CR and AL groups (*n* = 9–10 per group). **K** Eleven identified OTUs selected from total 20 OTUs by STAMP. Significance was calculated using the Kruskal–Wallis test (alpha diversity) and permutational multivariate analysis of variance test (beta diversity). ∗ *p* < 0.05, ∗  ∗ *p* < 0.01, ∗  ∗  ∗ *p* < 0.001, ∗  ∗  ∗  ∗ *p* < 0.0001

Diet is a major factor that shapes the gut microbiota and diet-microbiota interactions regulate the host metabolism through numerous mechanisms. It is a potential promising, novel, and cost-efficient therapeutic regimen for prevention of metabolic syndrome [14, 15]. Thus, we evaluated the how gut microbiota altered after short-term CR by 16 s rDNA gene amplicon sequencing in fecal samples collected from mice before and after CR. Pairwise orthogonal projection to latent structure-discriminant analysis (OPLS-DA) indicated a different profile of gut microbiota between AL and CR groups (Fig. 2A). As shown by principal coordinate analysis (PCoA) of the Bray–Curtis (Fig. 2B) and weighted_unifrac (Fig. 2C) distances, the gut microbiota overall structure revealed significant discriminative separation between the two groups, while alpha diversity did not change (Fig. 2D–G). At the phylum level, the microbiota community in male mice after CR showed lower *Bacteroidetes* abundance while higher *Firmicutes* abundance (Fig. 2H). Since *Firmicutes* could enhance energy harvest derived from diet [16], our results suggested the increase of *Firmicutes* following with restriction of caloric intake eventually contributes to making best use of limited food. We next employed random forests to build a classification model between the AL and CR mice. Totally, 20 OTUs were priority predictive for the gut microbiota differentiation between the CR and AL groups (Fig. 2I). Thirteen of the 20 OTUs decreased whereas the remaining 7 OTUs increased in the CR mice (Fig. 2J). These predicted OTUs revealed various changes even within the cogenetic family or genus after short-term CR. Of note, 3 OTUs (OTU3, OTU40, and OTU22) at the genus level of *Lactobacillus* and 2 OTUs (OTU204 and OTU105) in its species level of *reuteri* were all significantly enriched after short-term CR. Among the microbiota communities, genus *Lactobacillus* and species *reuteri* were shown the most predominant phylotype in CR group mice (Fig. 2K). In line with previous CR studies [3], our findings demonstrated that CR could shape the gut microbiota and *Lactobacillus* seem to be the prevalent predominant phylotype in CR mice.

### Short-term caloric restriction reprograms the transcriptome and modulates bile acid metabolism in liver

**Fig. 3:**
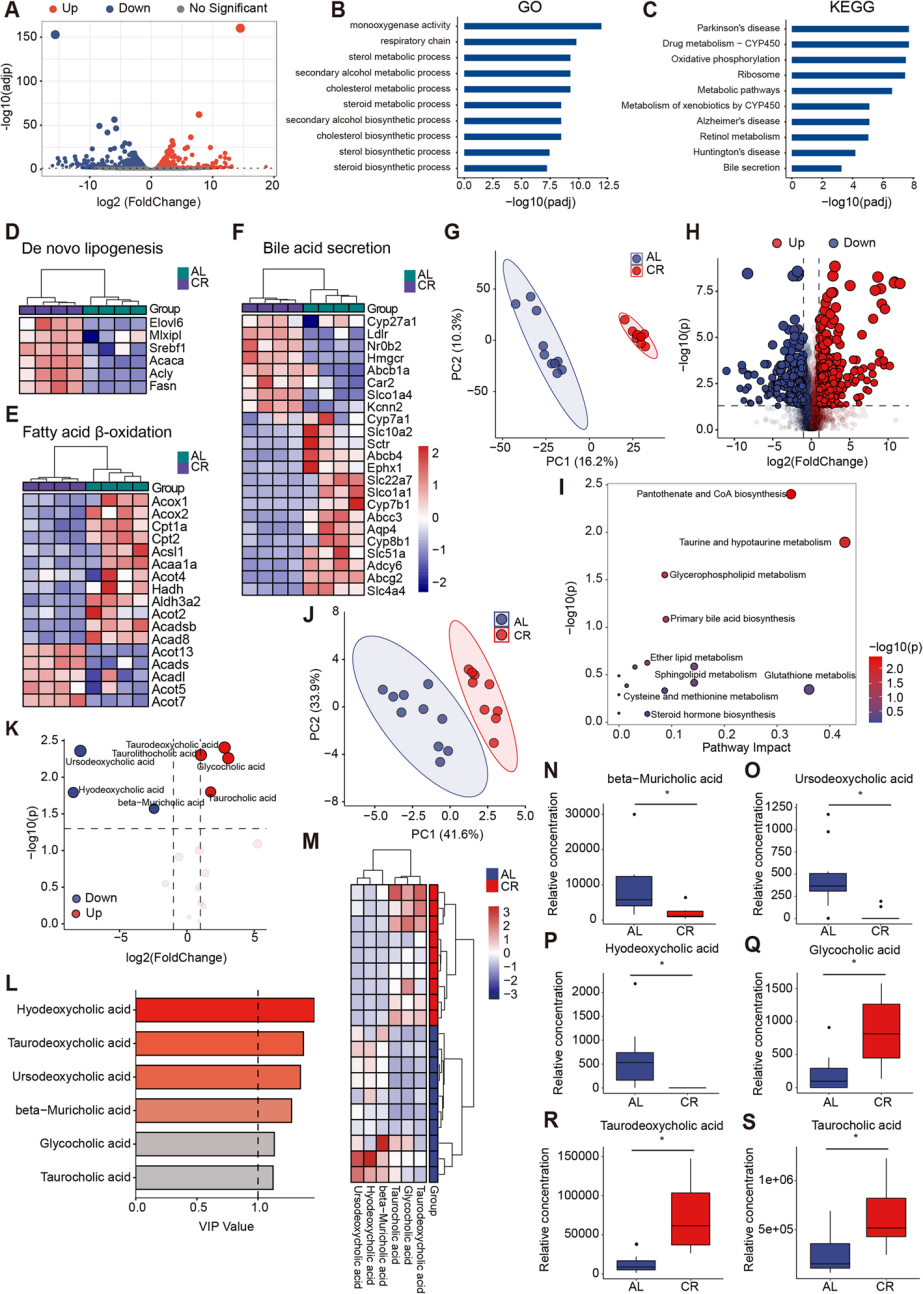
Short-term caloric restriction alters the transcriptome and modulates bile acid profile in liver.** A** Volcano plots of differentially expression genes (|fold change|> 2) between AL and CR groups (*n* = 4 per group). **B,C** GO and KEGG pathway analysis of differentially expression genes (both up and down) induced by AL or CR (*n* = 4 per group). **D–F** Heatmap of key genes involved in de novo lipogenesis, fatty acid β-oxidation, or in bile acid secretion (*n* = 4 per group). Each column contains data from an individual study animal and each row represents a gene. **G** OPLS-DA analysis of liver metabolites between AL and CR groups (*n* = 9–10 per group). **H** Volcano plots of differentially expression metabolites (|fold change|> 2) between AL and CR groups (*n* = 9–10 per group). **I** Metabolic enrichment pathway analysis based on the identified differentially expression metabolites. **J** OPLS-DA analysis of hepatic targeting bile acid metabolomics between AL and CR groups (*n* = 9–10 per group). **K** Volcano plots of differential bile acids (|fold change|> 2) between AL and CR groups (*n* = 9–10 per group). **L** Six identified bile acids selected by variable importance for the projection (VIP) between AL and CR groups (*n* = 9–10 per group, VIP > 1, *p* < 0.05). **M** Heatmap of six identified differential bile acids between AL and CR groups (*n* = 9–10 per group, VIP > 1, *p* < 0.05). **N–S** Relative concentrations of differential representative bile acids between AL and CR groups (*n* = 9–10 per group). Multiple testing correction was calculated. * *FDR* < 0.1, ** *FDR* < 0.05, *** *FDR* < 0.01 and **** *FDR* < 0.001 were determined statistically significant

To investigate the transcriptional response to CR, hepatic total RNA (4 livers per group) were prepared for RNA-seq. We identified 943 genes upregulated and 1555 genes downregulated via differentially expressed gene (DEG) analysis in liver (*N* = 31,058, *p* < 0.05; Fig. 3A, Additional file 2). Gene Ontology (GO) analysis suggested that differentially expressed genes (DEGs) were enriched in related metabolism pathways including sterol metabolic process, steroid metabolic process, and fatty acid metabolic process (Fig. 3B, Additional file 3). The Kyoto Encyclopedia of Genes and Genomes (KEGG) pathway analysis revealed DEGs participated in variety biological process including oxidative phosphorylation and bile secretion (Fig. 3C, Additional file 4). The dynamic balance of de novo lipogenesis (DNL) and fatty acid oxidation (FAO) is involved in lipid metabolism in metabolic diseases. In CR group, lipid metabolism was altered characterized by upregulation of genes mediating DNL (Fig. 3D) and of downregulation genes in FAO (Fig. 3E). Additionally, we also assessed alteration of genes involved in hepatic bile acid secretion, suggesting that CR regulated the bile acid metabolism (Fig. 3F).

To understand how CR affects the hepatic profile of small molecules, hepatic metabolome analysis was performed. Pairwise OPLS-DA indicated a different profile of liver metabolites after CR (Fig. 3G). Totally, 683 upregulated metabolites and 502 downregulated metabolites were identified (Fig. 3H, Additional file 5). Metabolic enrichment pathway analysis based on the identified differential metabolites showed that taurine and hypotaurine metabolism, primary bile acid biosynthesis, and steroid hormone biosynthesis were the key metabolic pathways altered (Fig. 3I).

Primary bile acids (BAs) are synthesized through cytochrome P450-mediated oxidation of cholesterol and conjugated to taurine (mainly in mice) or glycine (mainly in humans) in hepatocytes [17], which are important for host to maintain balanced lipid and carbohydrate metabolism, a healthy gut microbiota, insulin sensitivity, and innate immunity [17]. We further used UHPLC-MS/MS to quantitate 41 BAs in the liver from AL and CR mice. OPLS-DA analysis showed maximum discrimination between AL and CR mice (Fig. 3J). Totally, 17 BAs were qualitative identified (*N* = 41, VIP > 0; Fig. 3K, Additional file 1: Fig. S1, Additional file 6) and 6 BAs were significantly different between CR and AL groups (VIP > 1,p-value < 0.05; Fig. 3L,M). Of these, quantitative analysis indicated that the conjugated BAs glycocholic acid (GCA), taurolithocholic acid (TLCA), taurodeoxycholic acid (TDCA), taurocholic acid (TCA) were significantly elevated in the CR mice. The levels of unconjugated BAs such as hyodeoxycholic acid (HDCA), ursocholic acid (UCA), and beta-muricholic acid (beta-MCA) were significantly decreased while other bile acid levels were not changed in the CR group (FDR < 0.1, Fig. 3N-S, Additional file 1: Fig. S1). These results suggest that CR modulates the dynamics in BA composition along with increased concentrations of conjugated BAs in liver, which is consistent with the elevated conjugated BAs in improved metabolic phenotypes observed in previous research [18, 19].

### Short-term caloric restriction modifies the chromatin accessibility and enhancer landscape in liver

**Fig. 4:**
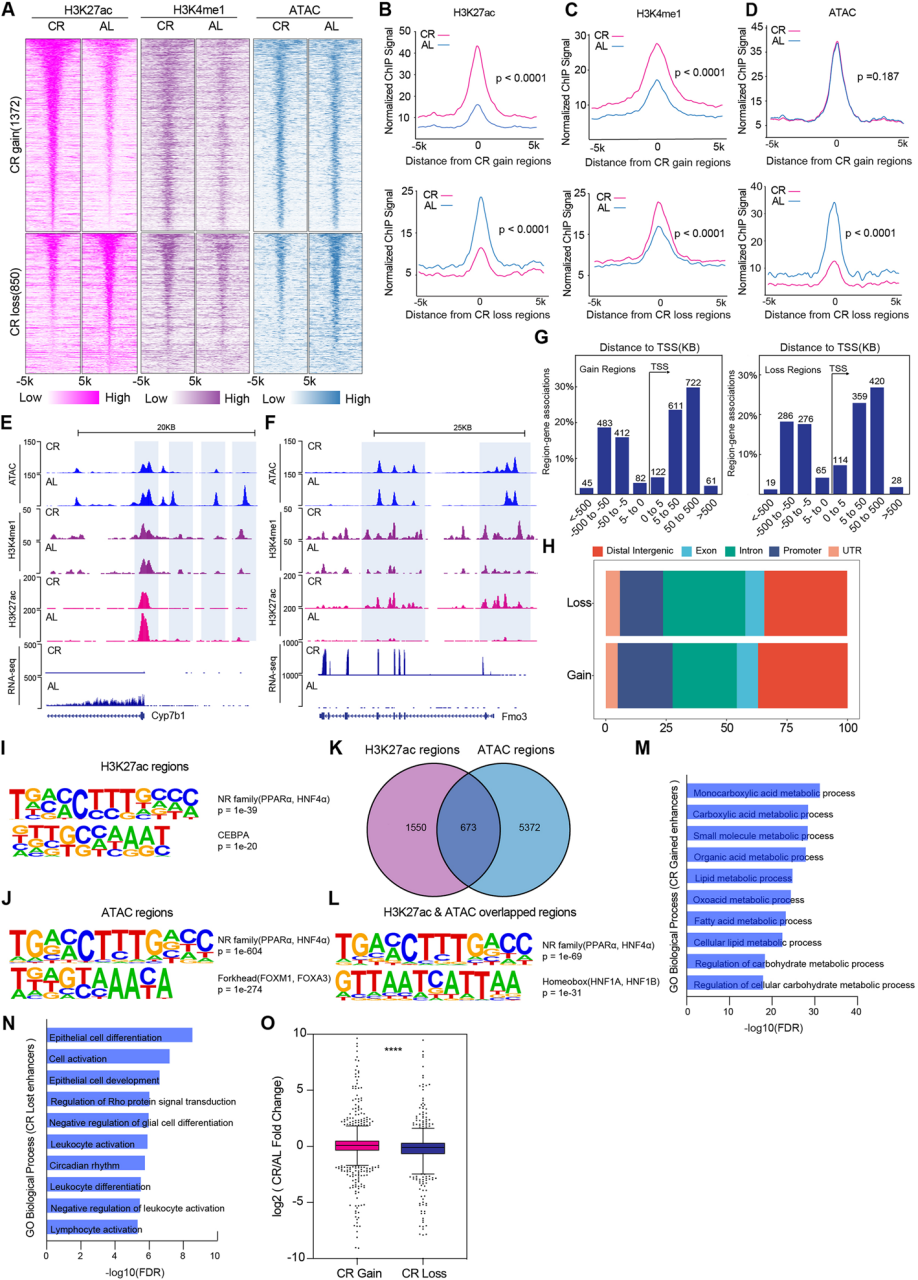
Short-term caloric restriction reprograms rewires the chromatin accessibility and enhancer network in liver.** A** Heatmap of H3K27ac and H3K4me1 differentially enriched regions and ATAC differentially chromatin accessibility in AL and CR groups (*n* = 2 per group). Each row in the heatmap represents an individual differentially locus. **B–D** Signals of H3K27ac and H3K4me1 differentially enriched regions and ATAC differentially chromatin accessibility in AL and CR groups (*n* = 2 per group). **E,F** Differentially representative enriched region of open chromatin, H3K27ac, H3K4me1, and gene expression levels in AL and CR groups (*n* = 2 per group). **E** Depicts the *Cyp7b1* locus which has lower chromatin accessibility, acetylation, and transcript levels in CR group. **F** Depicts the *Fmo3* locus which has higher acetylation, methylation, and transcript levels in CR group. **G** Distance to TSS of gain or loss H3K27ac regions between AL and CR groups (*n* = 2 per group). **H** Genomic features of the location of H3K27ac CR gain and loss peaks in AL or CR group (*n* = 2 per group). **I** Motif analysis of differentially H3K27ac enriched regions in AL or CR group (*n* = 2 per group). **J** Motif analysis of differentially open chromatin regions in AL or CR group (*n* = 2 per group). **K** Venn diagram of the differentially enriched H3K27ac regions and differentially open chromatin regions (*n* = 2 per group). **L** Motif analysis of differentially enriched H3K27ac regions and differentially open chromatin regions in AL or CR group (*n* = 2 per group). **M,N** GO pathway analysis of loci near CR gain or loss H3K27ac regions. **O** Gene expression in CR gain or loss H3K27ac regions (*n* = 2 per group). Significance was calculated using non-paired two-tailed Student’s *t* test. ∗ *p* < 0.05, ∗  ∗ *p* < 0.01, ∗  ∗  ∗ *p* < 0.001, ∗  ∗  ∗  ∗ *p* < 0.0001

Gene expression is regulated by regulatory elements, such as enhancers. To identify the regulatory elements responding to short-term CR, we profiled ATAC-seq and ChIP-seq in liver tissues. Since H3K27ac enrichment was correlated with active enhancer and gene transcription, we focused on H3K27ac and observed around 2000 loci with significantly differential enriched signal induced by CR (850 loss regions and 1372 gain regions (false discovery rate, *FDR < *0.05, foldchange > 2, Fig. 4A). Intriguingly, both CR gain or loss regions were pre-marked with H3K4me1 and distant from TSS regions (Fig. 4G), suggesting that these regions were enhancers (Fig. 4B–D). Sensitivity to transposition did not differ between CR and AL treatments at regions which gained H3K27ac, while loci losing H3K27ac also lost accessibility (Fig. 4D). Exemplar loci depicting accessibility, acetylation, and expression are presented in Fig. 4E,F. To annotate the location of H3K27ac CR gain and loss peaks in terms of genomic features, we used ChIPseeker to assign peaks to genomic annotation in the Distal Intergenic, Exon, Intron, Promoter, and UTR. We found that over 25% peaks were far from the gene promoters in CR gain and loss regions (Fig. 4H), which might be enhancers.

Enhancer regions usually harbor transcription factors (TFs) to enable selective gene expression by binding cognate cis-acting DNA sequences. To investigate differential TFs occupancy in our system, HOMER de novo motif algorithm [20] was used to determine which TF binding motifs are enriched at the differential acetylation enrichment loci and chromatin accessible regions. The top significantly enriched motifs (Fig. 4I) matched for NR family (PPARα and HNF4α), CEBPA across different open chromatin regions. Totally, 1074 regions with increased and 4971 regions with decreased chromatin accessibility (*FDR* < 0.05) were screened. Through HOMER, we also observed enrichment of HNF4α and PPARα binding motifs (Fig. 4J). We next performed overlap analysis between the differentially enriched H3K27ac regions and differentially open chromatin regions. Around 673 regions were overlapped and enriched with HNF4α binding sites (Fig. 4K,L). Consistent with our metabolome and transcriptome pathway results, GO analysis revealed several pathways enriched within genes around these loci, including metabolic process, such as lipid metabolic process, fatty acid metabolic process, and regulation of carbohydrate metabolic process (Fig. 4M,N). We next asked whether CR-remodeled enhancers contribute to the alteration of gene expression. Compared with the CR-lost enhancers, CR-gained enhancers were significantly linked with increases in gene expression (Fig. 4O). Taken together, our results demonstrated that CR altered chromatin modifications contributed to the transcriptional regulation of genes involved in metabolic process.

### Short-term caloric restriction affected HNF4α distribution in liver

**Fig. 5:**
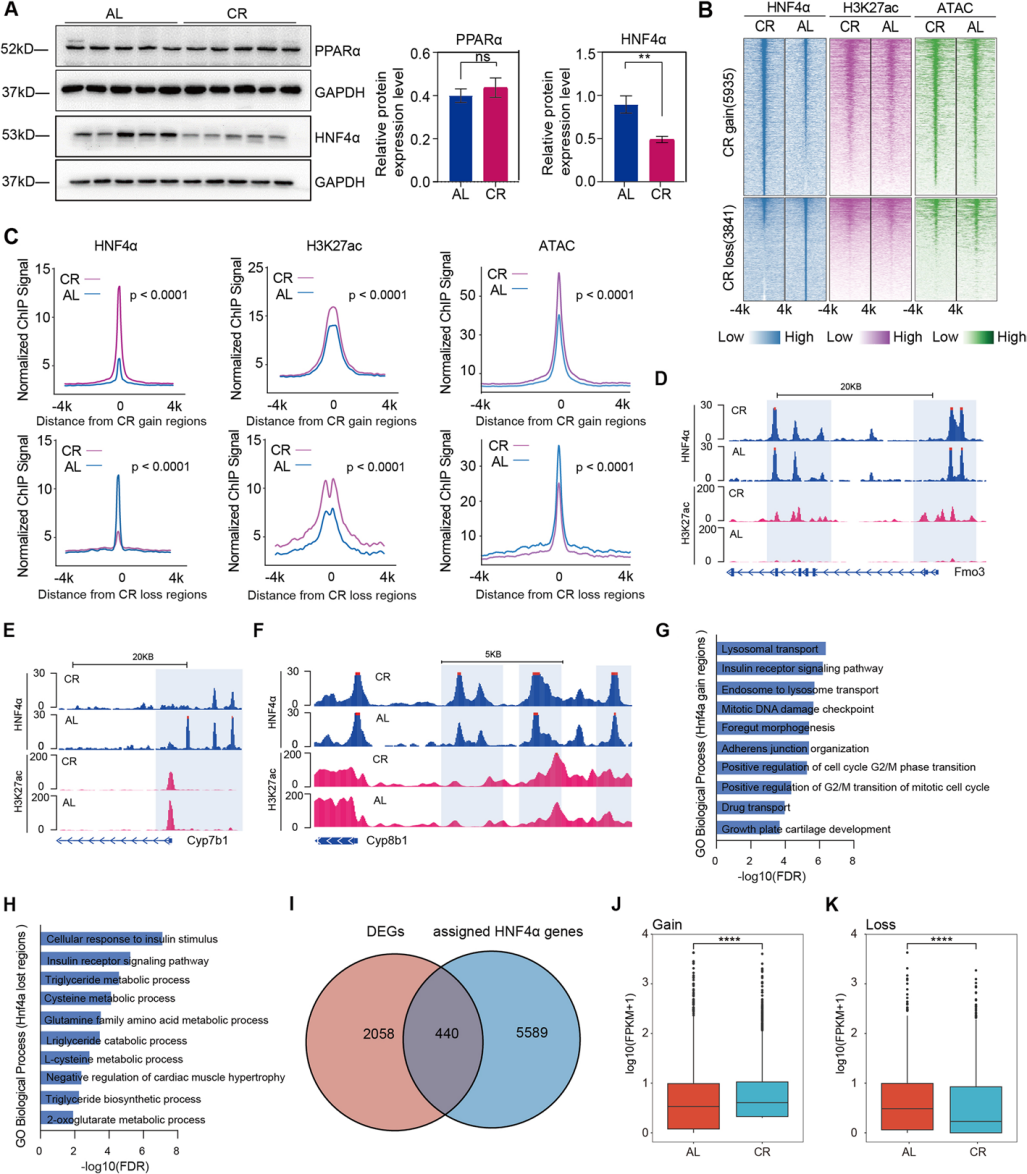
Short-term caloric restriction alters HNF4α distribution in liver.** A** Western blot analysis and quantitation of PPARα and HNF4α protein expression levels in AL and CR livers (*n* = 5 per group). **B** Heatmap of HNF4α, H3K27ac, and ATAC differentially CR gain or loss regions in AL and CR groups (*n* = 2 per group). **C** ChIP signals of HNF4α, H3K27ac, and ATAC in gain or loss region between AL and CR groups (*n* = 2 per group). **D** ChIP-seq of HNF4α and H3K27ac at the *Fom3* locus as a representative example (*n* = 2 per group). **E** ChIP-seq of HNF4α and H3K27ac at the *Cyp7b1* locus as a representative example (*n* = 2 per group). **F** ChIP-seq of HNF4α and H3K27ac at the *Cyp8b1* locus as a representative example (*n* = 2 per group). **G,H** GO pathway analysis of loci with differential HNF4α binding sites. **I** Venn diagram of DEGs (both up and down) induced by AL or CR and differential HNF4α peaks associated genes (*n* = 2 per group). **H** Expression levels of DEGs with CR-gained HNF4α peaks in AL and CR groups (*n* = 2 per group). Significance was calculated using non-paired two-tailed Student’s *t* test. ∗ *p* < 0.05, ∗  ∗ *p* < 0.01, ∗  ∗  ∗ *p* < 0.001, ∗  ∗  ∗  ∗ *p* < 0.0001

Binding sites of key transcription factors essential for fatty acid oxidation and bile acid biosynthesis were also enriched, including PPARα and HNF4α [21–24]. To this end, we used WB and qPCR to validate the expressions of the two signal-responsive TFs. We observed downregulation of HNF4α protein level in CR group despite no significant change in its mRNA level (Fig. 5A). This indicated that CR might alter the activation of HNF4α rather than its transcription. HNF4α, as a nuclear receptor, can bind to the genome to maintain chromatin structure and regulate gene expression in hepatocytes [25]. To investigate whether CR affect the distribution of HNF4α, we carried out HNF4α ChIP-seq and identified 5935 gain regions and 3841 loss regions (Fig. 5B). We observed that increased signal of HNF4α binding sites was accompanied by increased ATAC signal, and vice versa. Both regions were enriched for H3K27ac, which indicated that activated HNF4α was localized at activated promoter or enhancer loci (Fig. 5B,C). Paradigms of HNF4α binding regions with differential H3K27ac were shown in Fig. 5D–F. GO analysis showed that genes with differential HNF4α binding sites were involved in metabolism-related pathways, including insulin receptor signaling pathway, triglyceride metabolic process, and cellular response to insulin stimulus (Fig. 5G,H). We next leveraged our HNF4α ChIP-seq and RNA-seq data to determine whether differential HNF4α binding was accompanied by altered gene expressions. We assigned the differential HNF4α binding peaks to the nearest genes by ChIPseeker and observed around 440 DEGs were overlapped (*p*-value < 0.0001, Fig. 5I). DEGs with CR-gained HNF4α binding had significantly higher expression (*p*-value < 0.0001, Fig. 5J,K).

### Gut microbiota transplantation attenuated HFD-induced metabolic syndrome

**Fig. 6:**
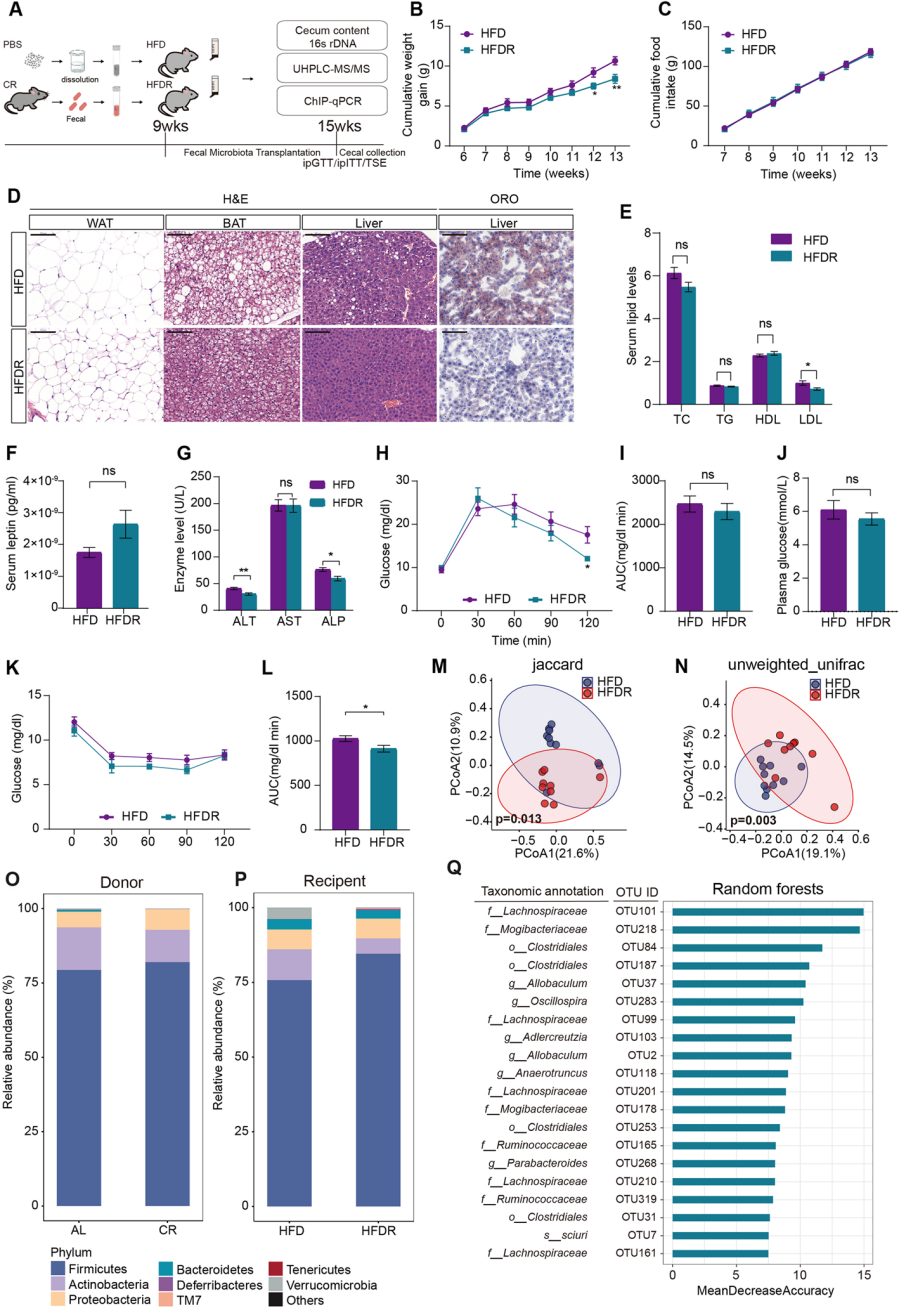
Gut microbiota transplantation alleviates HFD-induced metabolic syndrome and shapes gut microbiota composition.** A** The pattern diagram of gut microbiota transplantation in HFD mice. **B** Cumulative weight gain of male mice after gut microbiota transplantation (*n* = 8 per group). **C** Cumulative food intake of male mice after gut microbiota transplantation (*n* = 9–10 per group). **D** H&E staining of adipose tissue and liver in HFD and HFDR group (× 20 magnification; scale bar indicates 100 μm). BAT: Brown adipose tissue, WAT: White adipose tissue; Oil Red O staining of liver in HFD and HFDR group (× 20 magnification; scale bar indicates 100 μm). **E** Serum lipids levels of male mice from HFD or HFDR group (*n* = 6 per group). TC: total cholesterol, TG: triglyceride, LDL: low-density lipoprotein, HDL: high-density lipoprotein (*n* = 6 per group). **F** Serum leptin levels of male mice from HFD or HFDR group (*n* = 6 per group). **G** Serum ALT, AST, and ALP levels of male mice from HFD or HFDR group (*n* = 6 per group). **H,I** IPGTT tests in male mice from HFD and HFDR groups at 14 weeks and quantification of the AUC (*n* = 6 per group). **J** Plasma glucose levels of male mice in HFD and HFDR groups (*n* = 6 per group). **K,L** IPITT tests in mice from HFD and HFDR groups at 14 week and quantification of the AUC (*n* = 6 per group). **M,N** PCoA of the Jaccard and unweighted_unifrac distances at 15-week in HFD and HFDR group (*n* = 9–10 per group). **O,P** Phylum-level proportional abundance of cecal gut microbiota in donors (ALD and CRD) and recipients (HFD and HFDR) mice at 15 weeks (*n* = 9–10 per group). Each color represents a phyla. **Q** 20 OTUs associated with the differentiation of the gut microbiota between the HFD and HFDR groups based on random forests analysis (*n* = 9–10 per group). Significance was calculated using non-paired two-tailed Student’s *t* test and permutational multivariate analysis of variance test (beta diversity). ∗ *p* < 0.05, ∗  ∗ *p* < 0.01, ∗  ∗  ∗ *p* < 0.001, ∗  ∗  ∗  ∗ *p* < 0.0001

To investigate the central role of gut microbiota in regulating the beneficial adaptations of short-term CR on metabolic diseases, microbiota-depleted mice fed the high-fat diet (HFD, 60% kcal) were transplanted with CR microbiota (HFDR) by oral gavage (Fig. 6A). In line with previous results, donor mice fed CR diet decreased their weight compared to AL group (Additional file 1: Fig. S2A). Recipient mice (HFDR) receiving microbiota from CR mice (CR donor, CRD) had reduced weight compared to those receiving PBS (HFD) (Fig. 6B) with no changes in caloric uptake and food intake (Fig. 6C). WAT mass (epididymal and inguinal WAT) also decreased (Additional file 1: Fig. S2B). Histological analysis showed that adipocyte size in WAT and BAT in CR microbiota transplantation mice decreased (Fig. 6D). CR microbiota transplantation to obese mice also promoted a decrease in lipid deposits in hepatocytes (Fig. 6D). Serum LDL, ALT, and ALP were shown significant decreased in obese mice that received CR microbiota (Fig. 6E, G). No significant change was found in serum profile (TC, TG, HDL, and leptin), liver enzyme profile (AST), or energy expenditure in HFDR group mice (Fig. 6E–G, Additional file 1: Fig. S2C-H). We also performed GTT and ITT test and the ITT test results indicated that receiving microbiota from CR mice could improve insulin sensitivity in obese mice (Fig. 6H–L).

To decipher whether the CR microbiota directly contributes to these metabolic benefits, we used 16S rDNA sequencing to evaluate the microbiota composition in cecal samples in recipient and donor groups (Fig. 6M–R, Additional file 1: Fig. S3A-H). Principal coordinate analysis (PCoA) showed that Jaccard (Fig. 6M) and unweighted_unifrac (Fig. 6N) distances based on the OTU data were significantly discriminative between the HFD and HFDR groups. CR microbiota transplantation to obese mice did not alter the observed_otus (Additional file 1: Fig. S3I) and evenness (Additional file 1: Fig. S3J) indexes in the α-diversity of the gut microbiota. Donor and recipient cecal microbiomes analysis revealed that the recipients all changed in microbial diversity that was influenced by the diets (Fig. 6O,P). The phylum-level proportional abundance in HFDR group resembled that of donor mice following CR with high relative abundance of *Firmicutes* (Fig. 6O,P). Random forests analysis constructed a classification model between the HFD and HFDR groups. Totally, 20 predicted OTUs were highly identified for the differentiation of the gut microbiota between the HFD and HFDR groups (Fig. 6Q). Therefore, these results together uncover the core role of the functional gut microbiota, presumably through its metabolites, mediates a series of CR-induced metabolic improvements.

*Gut microbiota regulates BA metabolism *via* HNF4* α.

**Fig. 7:**
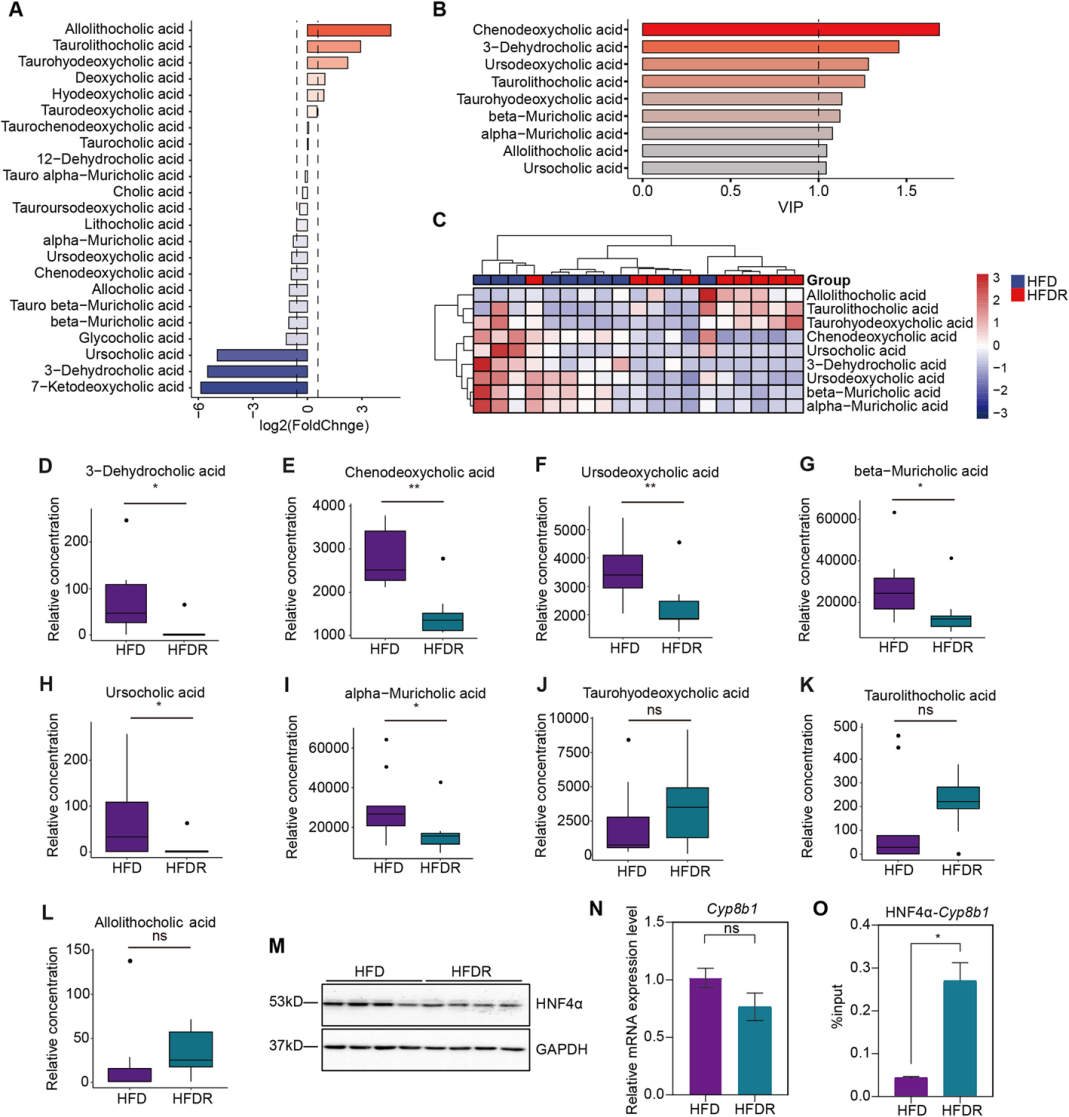
Gut microbiota shapes the hepatic HNF4α binding followed by altered *Cyp8b1* responsible for bile acid metabolism.** A** 23 identified differential bile acids (VIP > 0, *p*-value < 1) between HFD and HFDR groups (*n* = 9–10 per group). **B** 9 identified differential bile acids (VIP > 1, *p*-value < 1) between HFD and HFDR groups (*n* = 9–10 per group). **C** Heatmap of 9 identified differential bile acids (VIP > 1, *p*-value < 1) between HFD and HFDR groups (*n* = 9–10 per group). **D–L** Relative concentrations of differential representative bile acids between HFD and HFDR groups (*n* = 9–10 per group). **M** Western blot analysis of HNF4α in liver from HFD and HFDR groups (*n* = 4 per group). **N** Relative mRNA expression levels of *Cyp8b1* (*n* = 8 per group). **O** ChIP-qPCR analysis of *Cyb8b1* binding to HNF4α (*n* = 3 per group). Multiple testing correction was calculated. * *FDR* < 0.1, ** *FDR* < 0.05, *** *FDR* < 0.01 and **** *FDR* < 0.001 were determined statistically significant

To investigate whether gut microbiota transplantation also regulated BA metabolism, we further used targeted metabolomics approach to quantitate BAs from HFD and HFDR male mice. Totally, 23 BAs were qualitative identified (*N* = 41, VIP > 0; Fig. 7A, Additional file 7) and 9 BAs were different between HFDR and HFD groups with VIP > 1 (Fig. 7B,C). As shown in Fig. 7D–L, gut microbiota transplantation decreased the levels of unconjugated BAs such as 3-dehydrocholic acid (3-DHCA), chenodeoxycholic acid (CDCA), beta-muricholic acid (beta-MCA), ursodeoxycholic acid (UDCA), ursocholic acid (UCA), and alpha-muricholic acid (alpha-MCA) in the HFDR livers. Notably, decreased levels of beta-muricholic acid (beta-MCA) and ursodeoxycholic acid (UDCA) were found both in gut microbiota donor and recipient groups (*FDR* < 0.1, Fig. 7D–L, Fig. 3N–S, Additional file 1: Fig. S1, S4). These findings indicate that transferring gut microbiota can shape hepatic BA profiles resembling that observed in the CR mice.

We next wanted to ask whether gut microbiota remodeled the host epigenome to regulate BA metabolism. Consistent with previous findings, we found downregulation of HNF4α protein and no significant change in its mRNA level in HFDR (Fig. 7M), indicating HNF4α activation at the protein level. Next, we evaluated expression of *Cyp8b1*, which was downregulated in CR and involved in BA metabolism. We observed *Cyp8b1* was slightly decreased in HFDR, although it did not reach statistical significance (Fig. 7N). CYP8B1, the sterol 12α-hydroxylase, controls the key step to generate chenodeoxycholic acid (CDCA) [26]. Downregulation of CDCA (Fig. 7E) in HFDR might be caused by its downregulation. HNF4 α ChIP-seq data showed increased HNF4 α binding near *Cyp8b1* with decreased gene expression under CR (Fig. 5D, Fig. 3F). We next performed ChIP-qPCR to see whether downregulation of *Cyb8b1* was associated with HNF4 α binding. We observed HNF4 α binding near *Cyp8b1* was significantly increased in HFDR (Fig. 7O). These data indicate that gut microbiota transplantation is sufficient to recapitulate CR-induced BA metabolism and regulate gene expression through transcription factor binding.

## Discussion

Calorie restriction (CR) is as an effective and natural strategy for metabolic benefits and promotes lifespan in diverse species. There is substantial interest in elucidating the underlying molecular mechanisms that mediate the improved effects of a CR regimen since it is difficult for majority people with long-term adherence to a reduced-calorie diet. Numerous lines of evidence have identified relationships between gut microbiota and host diseases such as nonalcoholic liver disease, obesity, cardiovascular disorders, and malnutrition [27]. However, it is complicated to identify pivotal factors due to the complex functional interplays between host health, diet, and gut microbiota. Imbalance of the gut microbiota is shown to modify both host histone acetylation and methylation in various tissues [10]. In this study, we uncovered a unique paradigm of gut microbiota-dependent epigenomic plasticity of liver under calorie restriction.

**Fig. 8:**
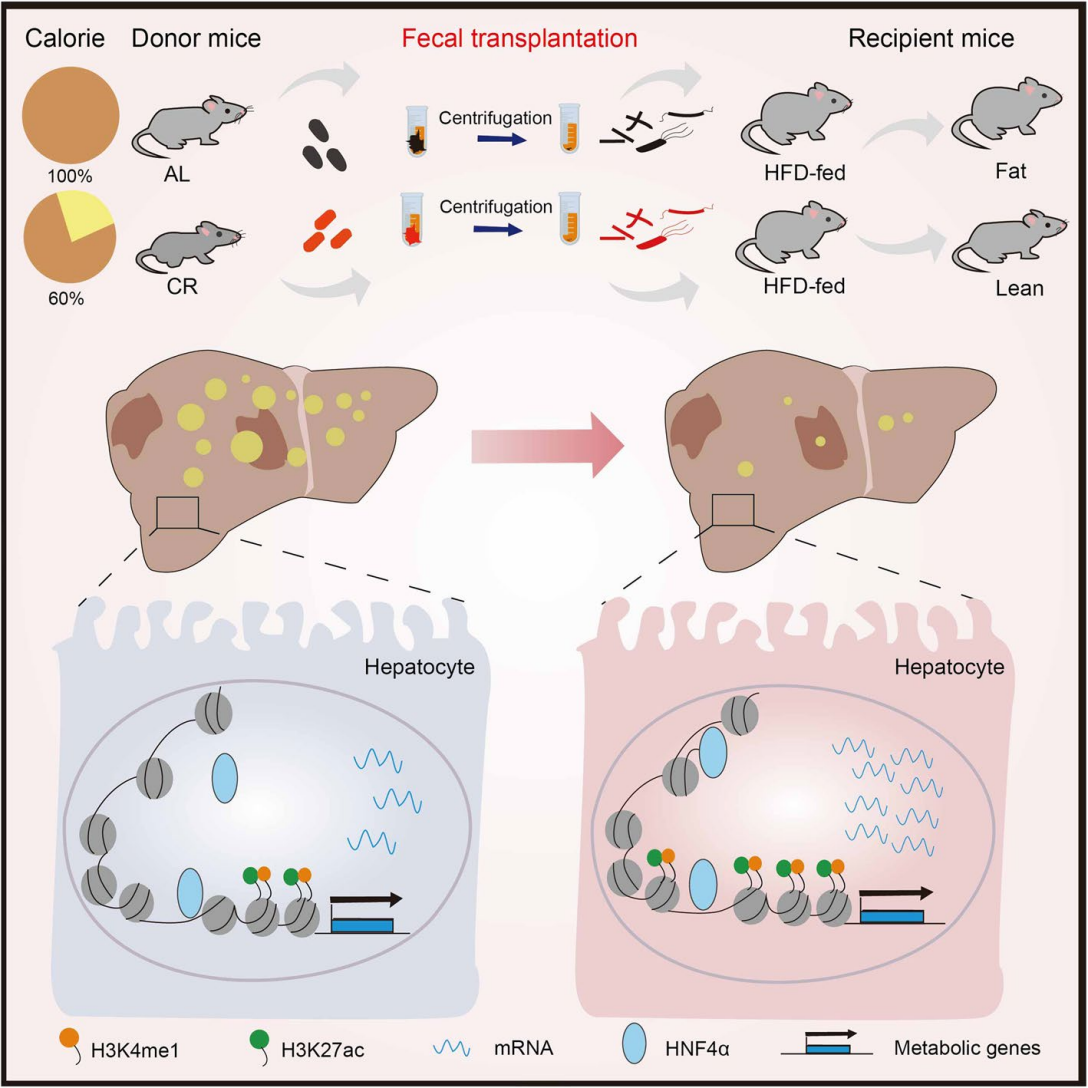
CR-induced microbiota shapes the hepatic epigenome coordinating altered Cyp8b1 responsible for bile acid metabolism. Short-term CR remodeled the hepatic enhancer landscape at genomic loci that were enriched for binding sites for signal-responsive transcription factors including HNF4α. Transferring CR gut microbiota into mice fed with an obesogenic diet recapitulated the features of CR-related bile acid metabolism along with attenuated fatty liver

Given that gut microbiota can influence host metabolism [4] and insulin sensitivity [28, 29], our hypotheses is that the beneficial improvements of CR are mainly owing to remodeling of gut microbiota, which is important in multiple metabolic adaptation phenotypes. The gut microbiota alteration during CR dictated the rhythm of the hepatic response as well as contributes to the fat burning, suppressed hepatic glucose production, and the multiple metabolic benefits observed during CR. In our study, CR-induced metabolic improvements could be partially recapitulated via FMT, suggesting the pivotal role of the CR-reshaped microbiota in promoting the overall metabolic improvements under CR (Fig. 8).

The gut microbiota was known to influence host intestinal homeostasis through systemic metabolism such as BA metabolism [9]. Here, we observed that CR influenced bile acid-related metabolic pathways such as taurine and hypotaurine metabolism, primary bile acid biosynthesis, and steroid hormone biosynthesis. The gut bacteria could modify BAs through both dehydroxylation to generate secondary bile acids and through deconjungation by bile salt hydrolase (BSH) enzymatic activity [30], an enzyme expressed in genus of *Lactobacillus* [31]. Genera *Lactobacillus* is acknowledged as one of the most frequently used probiotics, and its genus members enable anti-inflammatory effect and protect the function of host intestinal barrier [3]. Previous studies reported that *Lactobacillus* can regulate gut microbiota-modified deconjugation of primary BAs through bacterial transmembrane proton gradient-driven transportation, bacterial S-layer protein-mediated binding or activate intestinal nuclear receptor signaling pathway [32, 33]. In this study, the gut microbiota enriched in *Lactobacillus* was capable to modulate BA profiles. Also, we observed BA composition shift towards a profile resembling that found in microbiota-depleted animals reconstituted with microbiota from CR donors (Fig. 3, Additional file 1: Fig. S1, Fig. 7, Additional file 1: Fig. S4). Specifically, increased abundance of TLCA and decreased UDCA and β-MCA were noted in the liver. Previous study indicated secondary BAs (TLCA) increased more than primary BAs (UDCA and β-MCA) during CR [34]. Manipulating the gut microbiota is able to reverse the disturbance of host metabolism. Along these lines, HFD-fed animals (60%kcal) reconstituted with microbiota from CR donors had improved metabolic adaptation, potentially due to the BA composition alteration. Our current findings reinforce that functional flora may play a role in determining the BA composition.

An important physiological role of BAs is to regulate the process of emulsification and absorption of dietary lipids following their biliary secretion adaptation to food ingestion [35]. In the enterohepatic circulation, BAs are endogenous signaling factor that usually bind to its receptors to maintain BA homeostasis, regulate glucose and energy homeostasis, and mediate cholesterol and triglyceride metabolism [8, 36]. What is the consequence of these CR-induced microbiota and BA change? In our study, we observed the alterations of hepatic enhancer landscape, priming targets for signaling from microbiota and BAs. Within CR-related enhancers, the pivotal enriched motifs matched to NRs (PPARα and HNF4α). The protein level of HNF4α was decreased in CR group and HFD group with CR microbiota transfer (Figs. 5A, and 7M), thus we focused on HNF4α. HNF4α, an orphan member of the NR superfamily, was involved in metabolic regulation with the capacity of activating and repressing transcription [37, 38]. Consistent with other studies, we found BAs inhibited *Cyp8b1* expression and HNF4α protein level in both CR and HFDR group [23, 39]. HNF4α was reported to promote the *Cyb8b1* transcription through binding to its promoter region [23], while in the current study HNF4α binding near *Cyp8b1* promoter was increased. It might due to HNF4α’s dichotomous role to regulate gene expressions, not only direct promoting transcription, but also recruiting other factors to regulate gene expressions. Importantly, these current findings strongly reinforce our previous study pointing to the central role of HNF4α in regulating gene expression that was mediated by diet and functional flora [40].

## Conclusions

In summary, we have uncovered interactions between caloric restriction and microbiota and their effects on the hepatic epigenome and transcriptome, which regulate bile acid metabolism in host. Our results highlight the notion that gut microbiota can remodel the epigenome to fine-tune host metabolic adaptations.

## Methods

### Mice and antibiotics treatment

All mouse experiments were performed following all regulatory standards approved by the Nanjing Medical University (NJMU) Institutional Animal Care and Use Committee (IACUC). Five-week-old C57BL/6 male mice were obtained and acclimated under a cycle of 12-h dark/light at a temperature of 22 °C ± 3 °C for one additional week. Before caloric restriction treatment, mice were fed on a normal chow diet (3.53 total kcal/gm; 12 kcal% fat, 20.6 kcal% protein, 67.4 kcal% carbohydrates, SWS9102, Xietong, China) for 1 week. Subsequently, the mice were randomly divided into two study groups (10 mice per group) and fed ad libitum with a low-fat diet (LFD) (3.85 total kcal/gm; 10 kcal% fat, 20 kcal% protein, 70 kcal% carbohydrates, Xietong, China) or a 40% calorie restriction diet (CRD) (3.75 total kcal/gm; 17 kcal% fat, 33 kcal% protein, 50 kcal% carbohydrates, Xietong, China) for up to 12 weeks (Additional file 1: Table S2). Caloric restriction group mice were fed daily at 5PM with 40% less food than that the ad libitum fed mice. All mice were singly housed to ensure each CR group mouse can get 60% calorie of the AL group.

To eradicate gut microbiota, the mice were fed ad libitum with drinking water containing 1 mg/mL Ampicillin, 1 mg/mL Neomycin, 1 mg/mL Streptomycin, 1 mg/mL Ciprofloxacin, and 0.5 mg/mL Vancomycin (all antibiotics were from MedChemExpress USA) added for 1 week. Recipient male mice were used with gastric gavage of oral antibiotics and the antibiotics were renewed every day. About 100 mg fresh stool was collected from either caloric restricted male mice (donor mice) or ad libitum male mice and then resuspended, homogenized, and centrifuged to rule out debris. Recipient male mice were inoculated by oral gavage with 100 μl of the supernatant 4 times per week for 5 weeks. During gut microbiota transplantation, the recipient mice were fed ad libitum with LFD or high-fat diet (HFD) (5.24 total kcal/gm; 60 kcal% fat, 20 kcal% protein, 20 kcal% carbohydrates, D12492, Xietong, China).

### Glucose-insulin tolerance tests

For intraperitoneal glucose tolerance tests (IPGTTs), fasted mice (12 h) were administered with an intraperitoneal (i.p.) injection of glucose (2 g/kg) at 12 weeks. Intraperitoneal insulin tolerance tests (IPITTs) were tested 2 days later. Fasted mice (6 h) were injected intraperitoneally with 0.75 IU/kg of insulin (0.75 U/kg). Blood glucose was recorded at 0, 30, 60, 90, and 120 min applying the ACCUCHEK active glucometer (Roche).

### Energy metabolism measurements

To monitor the effects of CR on metabolic parameters, 13-week-old mice (6 animals per group) were adapted for 2 days and tested for 3 days in the Department of Animal Core Facility of Nanjing Medical University. Mice were free to food and water for the whole test duration. Oxygen consumption (VO_2_), respiratory exchange ratio (RER) carbon dioxide release (VCO_2_), and energy expenditure (EE) were analyzed using a combined TSE phenoMaster system (TSE Phenomaster, Germany) with a ~ 0.25 l/min air flow rate and a 39-min record cycle in our study. The RER was evaluated by the ratio of VCO_2_/VO_2_ and EE was normalized with body weight.

### Plasma content analysis

Blood samples were collected by the intraocular vein from live mice fasted overnight at 15 weeks. The serum layer was collected and its levels of alkaline phosphatase (ALP), alanine transaminase (ALT), and aspartate transaminase (AST), total cholesterol (TC), high-density lipoprotein (HDL), triglycerides (TGs), low-density lipoprotein (LDL), and glucose were measured in the Department of Animal Core Facility of Nanjing Medical University.

### Histology and lipid staining analysis

Livers and adipose tissues were excised and fixed in 4% paraformaldehyde for 24 h. Then paraffin-embedded tissue Sects. (5 μm) were stained with hematoxyline and eosin (H&E) for structural analysis. Ice-cold paraformaldehyde-fixed frozen sections from mice were stained with Oil Red O (ORO). Light microscope (Olympus, Tokyo, Japan) was applied to further observe the staining images.

### 16S rDNA gene sequencing and data analysis

Stool and cecal content were obtained fresh from study mice in a sterile environment and extracted bacterial DNA isolation using QIAamp DNA Stool mini kit (Qiagen, Germany) based on the provided instructions. PCR reactions were applied to amplify hypervariable regions 3 and 4 (V3-V4) of the 16S rDNA gene for sequencing based on a MiSeq platform (Illumina Inc., San Diego, USA). Amplicons were sequenced on the MiSeq PE250 platform.

Sequence quality control was analyzed by QIIME2 with Greengenes release database (version 13.8). Operational taxonomic units (OTUs) were obtained using a similarity threshold against the Greengenes database at 97%. OTUs were normalized by sequencing depth and alpha diversity was also calculated by QIIME2. Weighted UniFrac and principal coordinate analysis (PCoA) were applied to characterize beta diversity. Random forests analysis was applied to establish the classification model using the ‘randomForest’ R package with all default settings [3]. OTU was assigned mean decrease in accuracy (MDIA) value as its importance score and the MDIA score of OTUs at least 0.001 were then picked as highly predictive to distinguish gut microbiota between the AL and CR groups. Relative proportions of predicted OTUs were compared by statistical hypothesis tests with *p*-value corrected in STAMP 2.1.3.

### RNA-seq and data analysis

The total RNA was isolated from AL or CR mice livers at 12 weeks. RNA quality test was evaluated by the Bioanalyzer 2100 system (Agilent Technologies, CA, USA). RNA sequencing was performed in Novogene and being sequenced based on the Illumina NovaSeq 6000 to generate read alignments with mm10.

Raw counts mapped to genes were obtained from featureCounts (v1.5.0-p3), normalized, and analyzed using DEseq2. The Benjamini and Hochberg’s method was used to adjust the false discovery rate (FDR) used to corrected the *p*-value. *FDR* < 0.05 was identified to screen the differentially expressed genes.

### Metabolomic analysis and BAs analysis

Fifty milligrams liver samples were homogenized and sonicated on ice for 5 min. The liver samples were incubated for 1 h at − 20 ℃ and centrifuged at 4 ℃ (11,000 rpm, 15 min). 0.5 mL supernatant was subjected to a fresh glass vial for LC–MS/MS analyses [41]. Targeted bile acid analysis was conducted using an Agilent 1290 Infinity series UHPLC System (Agilent Technologies).

### ATAC-seq and data analysis

ATAC-seq libraries were obtained from frozen liver tissues using the Omni-ATAC protocol as described with slight modifications [42]. Liver tissue was homogenized by Dounce (Wheaton Dounce, 1 ml, loose) with cold PBS. The homogenized solution was filtered with a 70-μM cell strainer (BD Falcon). Pelleted hepatocytes were resuspended in transposition mixture (2 × TD buffer, transposase (100 nM final), 1% digitonin, 10% Tween-20). The sample reaction was incubated at 37 °C for 30 min in a thermomixer. The transposed fragments were amplified with custom primers in 9–12 cycles and purified by Agencourt AMPure XP beads (Beckman Coulter). Hepatic ATAC-seq libraries were sequenced on Illumina NextSeq 500 platform. Bowtie2 was then used to map sequenced reads to the mouse genome and removed if they were duplicated, unmapped, or mapped to mitochondrial genome [43]. DiffBind package with FDR values < 0.05 was used to identify the differentially enriched ATAC loci.

### ChIP-seq and data analysis

Liver samples were obtained for ChIP-seq analysis according to our previous study [40]. Briefly, homogenized livers tissue was fixed with 1% formaldehyde. Cross-linking was quenched with 0.125 M glycine. Chromatin was sonicated to obtain ~ 200 bp fragments for immunoprecipitation using Covaris. H3K27ac (ab4729, Abcam), H3K4me1 (ab8895, Abcam), and HNF4α (ab41898) antibodies (Additional file 1: Table S3) were used and the protein–DNA complexes were eluted, reverse crosslinked, and purified. One nanogram of ChIPed DNA was prepared applying NEXTflex Rapid Illumina DNA-Seq Library Prep Kit (Bio Scientific), and the ChIP-seq libraries were using Illumina Nextseq 500 platform to sequence.

Raw sequenced reads [43] were filtered mapped to the mouse genome (mm9). SICER (v1.1) was used to peak calling with the following parameters of H3K27ac. DiffBind package with FDR values < 0.01 and fold change > 2 were used to identify the differentially enriched H3K27ac loci. The HOMER package was applied to peak calling for HNF4α with “-style factor” and find Motifs. For the HNF4α differential binding analysis, HOMER was used with default setting.

#### Real-time PCR

Total RNA was extracted using TRIzol reagent (Life Technologies, USA), and mRNA levels were tested by qPCR using SYBR Green (Roche, Basel, Switzerland). Normalized RNA concentrations were converted to cDNA and mRNA expression used glyceraldehyde-3-phosphate dehydrogenase (*Gapdh*) control to normalize. A subset of the differentially expressed genes in RNA-seq profiling were tested by qPCR and primers sets were designed using primer bank (Additional file 1: Table S4). The primers of ChIP-qPCR are demonstrated in Additional file 1: Table S5.

### Statistical analysis

Data in this study were analyzed by Prism 8 (GraphPad Software), and statistical significance was determined using two-tailed unpaired Student’s *t* test or Mann–Whitney test. Multiple comparisons were done using one-way analysis of variance (ANOVA) test. The statistical significance of the overlap was determined by chi-squared test. *p < 0.05, **p < 0.01, ***p < 0.001, and ****p < 0.0001 were determined statistically significant. Multiple testing correction was calculated. * *FDR* < 0.1, ** *FDR* < 0.05, *** *FDR* < 0.01, and **** *FDR* < 0.001 were determined statistically significant.

## Supplementary Information

**Additional file 1: Fig. S1.** Relative concentrations of identified bile acids in AL and CR groups. **Fig.S2.** The phenotypes of HFD male mice after CR gut microbiota transplantation. **Fig. S3.** Cecal gut microbiota composition alterations in donor groups and recipient groups. **Fig. S4.** Relative concentrations of identified bile acids in HFD and HFDR groups. **Table S1.** The caloric consumption in the study. **Table S2.** The components of the diet sused in the study. **Table S3.** Primary antibodies used in this study. **Table S4.** Sequences of primers for RT-qPCR. **Table S5.** Sequences of primers for ChIP-qPCR. 

**Additional file 2.** Differential expressed genes in livers (AL vs CR).

**Additional file 3.** Top20 GO enrichment analysis of hepatic DEGs.

**Additional file 4.** Top20 KEGG pathway analysis of hepatic DEGs.

**Additional file 5.** Differentially expressed metabolites in livers.

**Additional file 6.** Differentially identified BAs in livers (AL vs CR).

**Additional file 7.** Differentially identified BAs in livers (HFD vs HFDR).

**Additional file 8.** Review history.

## Data Availability

The datasets analyzed during the current study are available from the corresponding author on reasonable request. The raw sequence data reported in this study have been deposited in the Genome Sequence Archive [44] in National Genomics Data Center [45], China National Center for Bioinformation / Beijing Institute of Genomics, Chinese Academy of Sciences (GSA: CRA010174) [43] that are publicly accessible at https://ngdc.cncb.ac.cn/?lang=en.
