# Supplementary material for: Caloric restriction remodels the hepatic chromatin landscape and bile acid metabolism by modulating the gut microbiota
Source: Genome Biol. 2023 Apr 30;24:98. doi: 10.1186/s13059-023-02938-5 (PMC10150505; doi:10.1186/s13059-023-02938-5)
Supplement: Supplementary file 1 — Additional file 1: Fig. S1. Relative concentrations of identified bile acids in AL and CR groups. Fig.S2. The phenotypes of HFD male mice after CR gut microbiota transplantation. Fig. S3. Cecal gut microbiota composition alterations in donor groups and recipient groups. Fig. S4. Relative concentrations of identified bile acids in HFD and HFDR groups. Table S1. The caloric consumption in the study. Table S2. The components of the diet sused in the study. Table S3. Primary antibodies used in this study. Table S4. Sequences of primers for RT-qPCR. Table S5. Sequences of primers for ChIP-qPCR. [file 13059_2023_2938_MOESM1_ESM.docx]

**Fig. S1.**


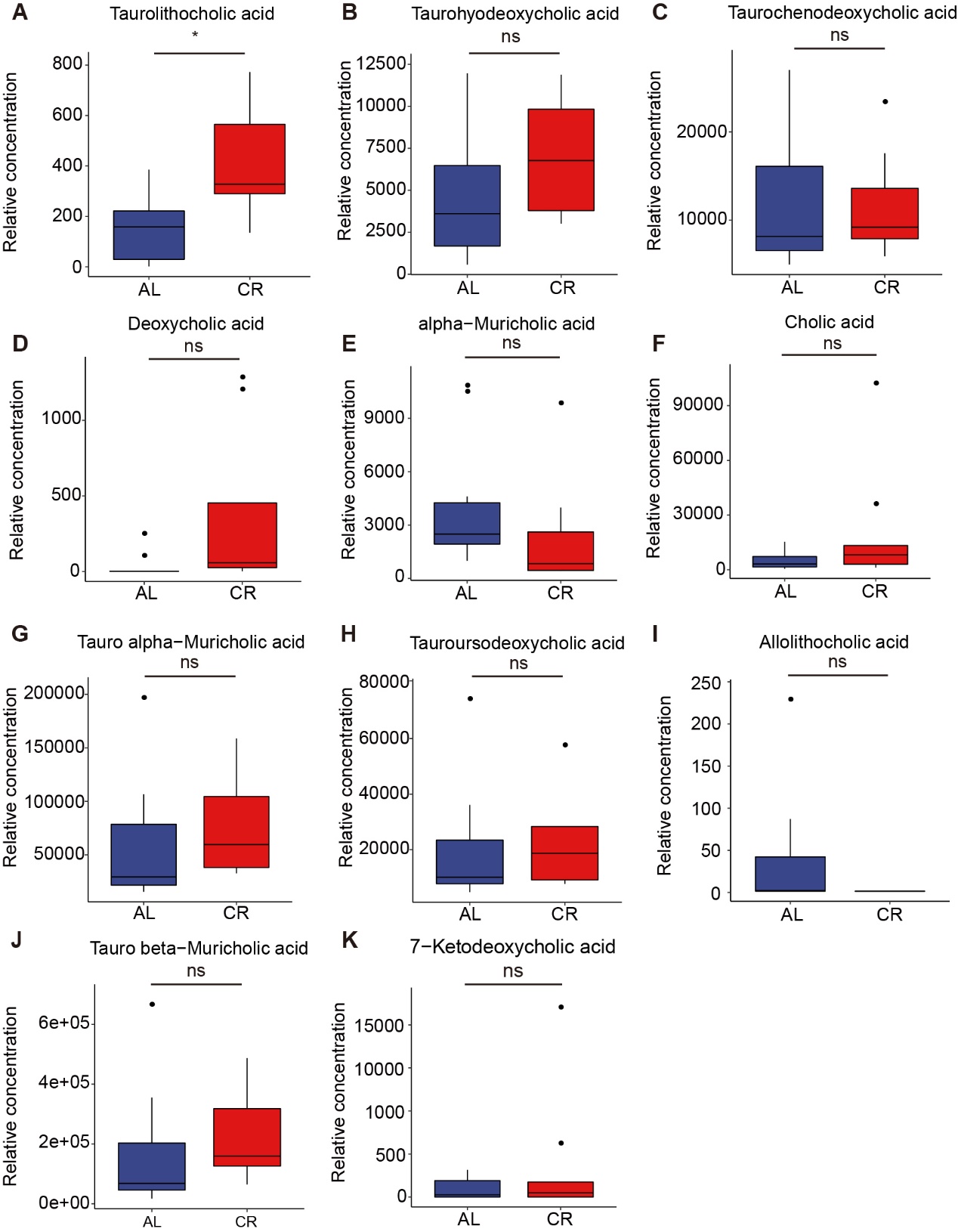


**Relative concentrations of identified bile acids in AL and CR groups.** (**A**) Relative concentration of taurolithocholic acid in AL and CR group (n=9-10 per group). (**B**) Relative concentration of allolithocholic acid in AL and CR group (n=9-10 per group). (**C**) Relative concentration of 7-Ketodeoxycholic acid in AL and CR group (n=9-10 per group). (**D**) Relative concentration of alpha-Muricholic acid in AL and CR group (n=9-10 per group). (**E**) Relative concentration of cholic acid in AL and CR group (n=9-10 per group). (**F**) Relative concentration of deoxycholic acid in AL and CR group (n=9-10 per group). (**G**) Relative concentration of tauro alpha-Muricholic acid in AL and CR group (n=9-10 per group). (**H**) Relative concentration of tauroursodeoxycholic acid in AL and CR group (n=9-10 per group). (**I**) Relative concentration of taurohyodeoxycholic acid in AL and CR group (n=9-10 per group). (**J**) Relative concentration of tauro beta-Muricholic acid in AL and CR group (n=9-10 per group). (**K**) Relative concentration of taurochenodeoxycholic acid in AL and CR group (n=9-10 per group). Multiple testing correction was calculated. * *FDR* < 0.1, ** *FDR* < 0.05, *** *FDR* < 0.01 and **** *FDR* < 0.001 were determined statistically significant.

**Fig. S2.**


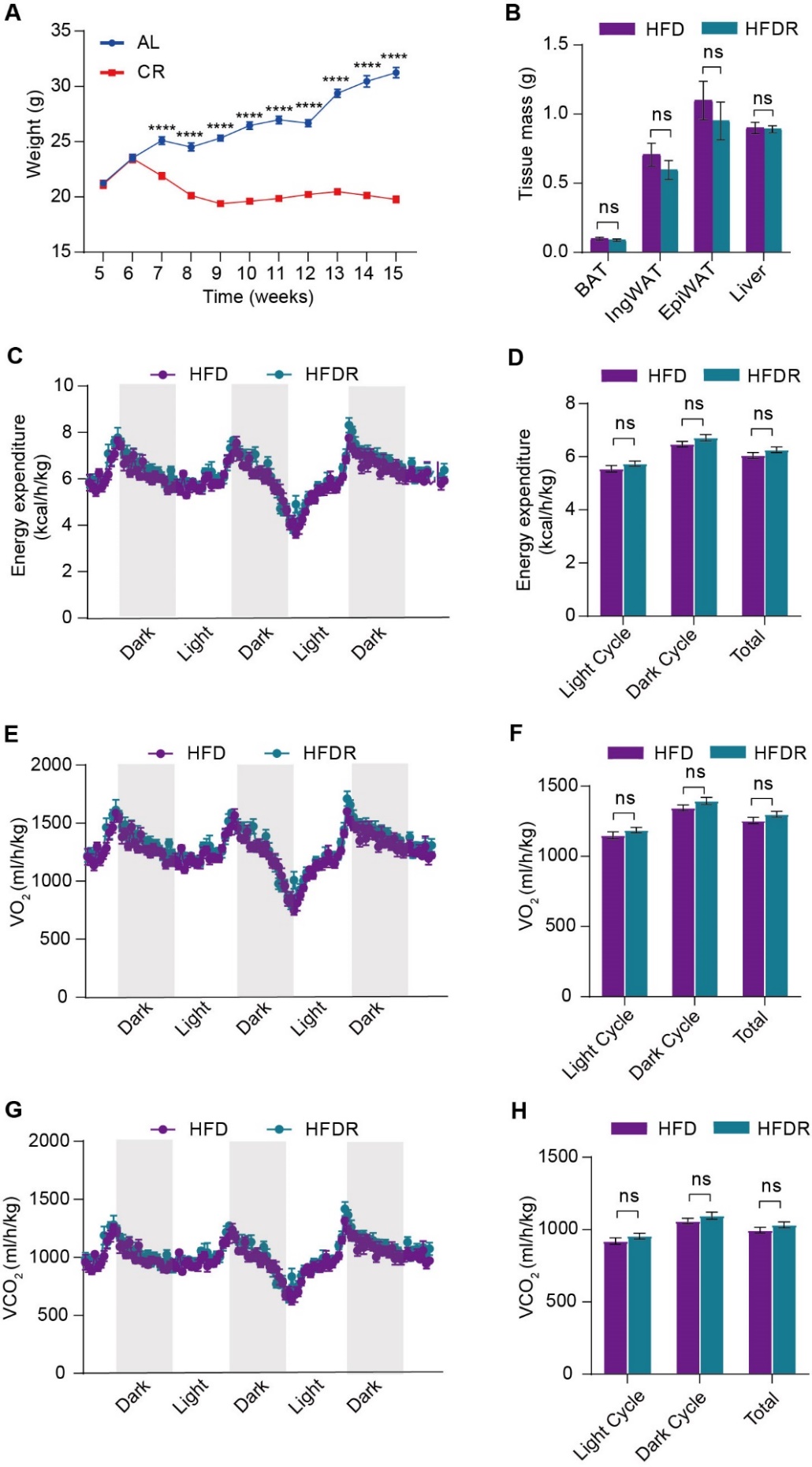


**The phenotypes of HFD male mice after CR gut microbiota transplantation.** (**A**) Body weight of male mice after AL or CR treatment (n=10 per group). (**B**) Brown adipose tissue (BAT), inguinal adipose tissue (IngWAT), epididymal adipose tissue (EpiWAT), and liver mass of male mice in HFD or HFDR groups (n= 8-10 per group). (**C-H**) TSE phenoMaster cages analysis of oxygen energy expenditure (EE), consumption rate (VO_2_), and carbon dioxide production (VCO_2_), of male mice in the HFD or HFDR groups (n=6 per group). Significance was calculated using non-paired two-tailed Student’s t test. ∗*p* < 0.05, ∗∗*p* < 0.01, ∗∗∗*p* < 0.001, ∗∗∗∗*p* < 0.0001.

**Fig. S3.**


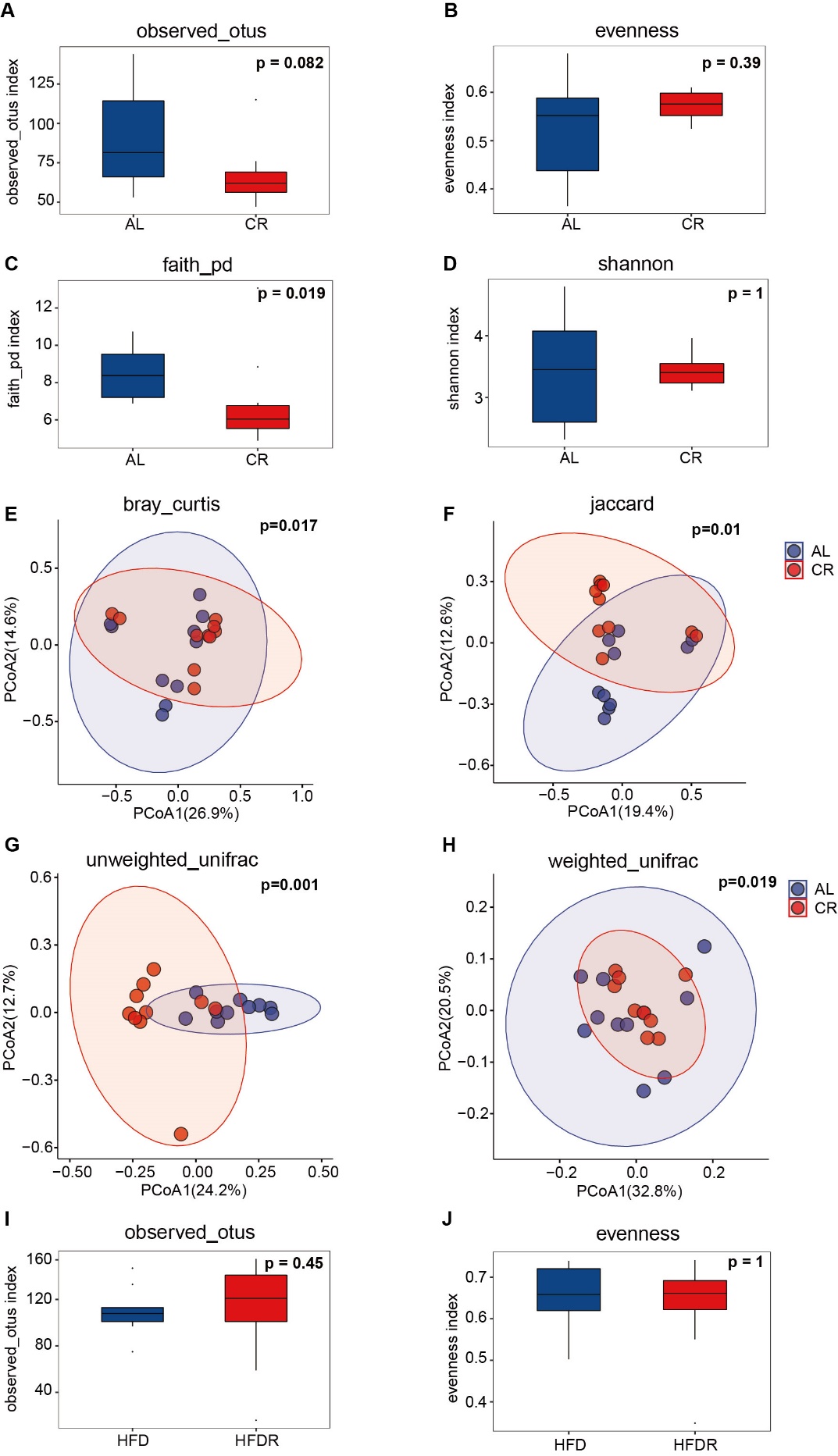


**Cecal gut microbiota composition alterations in donor groups and recipient groups.** (**A-D**) Indexes of observed_otus, evenness, faith_pd, Shannon of cecal samples from AL or CR group at 15-week (n=10 per group). (**E-H**) Principal coordinate analysis (PCoA) of the bray-curtis and weighted_unifrac distances of cecal samples from AL or CR group at 15-week (n=10 per group). (**I-J**) Indexes of observed_otus and evenness of cecal samples from HFD or HFDR group at 15-week (n=9-10 per group). Significance was calculated using Kruskal-Wallis test (alpha diversity) and permutational multivariate analysis of variance test (beta diversity). ∗*p* < 0.05, ∗∗*p* < 0.01, ∗∗∗*p* < 0.001, ∗∗∗∗*p* < 0.0001.

**Fig. S4.**


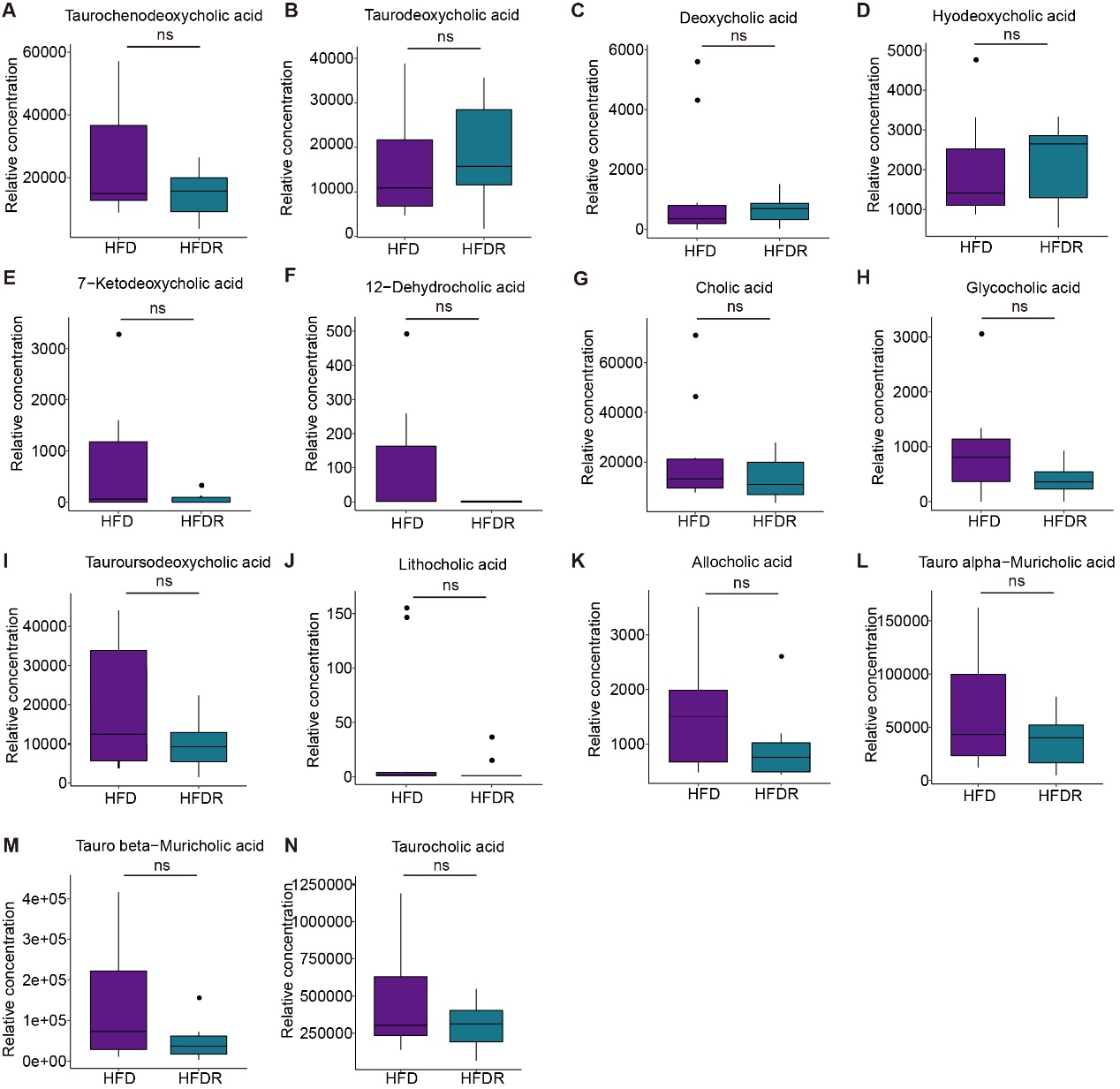


**Relative concentrations of identified bile acids in HFD and HFDR groups.** (**A**) Relative concentration of taurochenodeoxycholic acid in HFD and HFDR groups (n=9-10 per group). (**B**) Relative concentration of taurodeoxycholic acid in HFD and HFDR groups (n=9-10 per group). (**C**) Relative concentration of deoxycholic acid in HFD and HFDR groups (n=9-10 per group). (**D**) Relative concentration of hyodeoxycholic acid in HFD and HFDR groups (n=9-10 per group). (**E**) Relative concentration of 7-ketodeoxycholic acid in HFD and HFDR groups (n=9-10 per group). (**F**) Relative concentration of 12-dehydrocholic acid in HFD and HFDR groups (n=9-10 per group). (**G**) Relative concentration of cholic acid in HFD and HFDR groups (n=9-10 per group). (**H**) Relative concentration of glycocholic acid in HFD and HFDR groups (n=9-10 per group). (**I**) Relative concentration of tauroursodeoxycholic acid in HFD and HFDR groups (n=9-10 per group). (**J**) Relative concentration of lithocholic acid in HFD and HFDR groups (n=9-10 per group). (**K**) Relative concentration of allocholic acid in HFD and HFDR groups (n=9-10 per group). (**L**) Relative concentration of tauro alpha-Muricholic acid in HFD and HFDR groups (n=9-10 per group). (**M**) Relative concentration of tauro beta-Muricholic acid in HFD and HFDR groups (n=9-10 per group). (**N**) Relative concentration of taurocholic acid in HFD and HFDR groups (n=9-10 per group). Multiple testing correction was calculated. * *FDR* < 0.1, ** *FDR* < 0.05, *** *FDR* < 0.01 and **** *FDR* < 0.001 were determined statistically significant.

**Table S1: The caloric consumption in the study.**

| **Group#** | **Control Group** | | **CR Group** | |
| --- | --- | --- | --- | --- |
|  | **gm (%)** | **Kcal (%)** | **gm (%)** | **Kcal (%)** |
| Protein | 19.2 | 20 | 31.3 * 0.6 = 18.8 | 33 * 0.6 = 19.8 |
| Carbohydrate | 67.3 | 70 | 46.9 * 0.6 = 28.1 | 50 * 0.6 = 30 |
| Fat | 4.3 | 10 | 6.9 * 0.6 = 4.1 | 17 * 0.6 = 11.2 |
| Total |  | 100 |  | 100 * 0.6 = 60 |
| **kcal/gm** | **3.85** |  | **3.75** | **kcal/gm** |

**Table S2: The components of the diets used in the study.**

| **Product#** | **Control Diet** | | **Caloric Restriction Diet** | |
| --- | --- | --- | --- | --- |
|  | **gm (%)** | **Kcal (%)** | **gm (%)** | **Kcal (%)** |
| Protein | 19.2 | 20 | 31.3 | 33 |
| Carbohydrate | 67.3 | 70 | 46.9 | 50 |
| Fat | 4.3 | 10 | 6.9 | 17 |
| Total |  | 100 |  | 100 |
|  |  |  |  |  |
| **Ingredient** | **gm** | **kcal** | **gm** | **kcal** |
| Casein, 80 Mesh | 200 | 800 | 200 | 800 |
| L-Cystine | 3 | 12 | 3 | 12 |
|  |  |  |  |  |
| Corm Starch | 315 | 1260 | 110 | 440 |
| Maltodextrin 10 | 35 | 140 | 35 | 140 |
| Sucrose | 350 | 1400 | 149.3 | 597 |
|  |  |  |  |  |
| Cellulose, BW200 | 50 | 0 | 50 | 0 |
|  |  |  |  |  |
| Soybean Oil | 25 | 225 | 25 | 225 |
| Lard | 20 | 180 | 20 | 180 |
|  |  |  |  |  |
| Mineral Mix S10026 | 10 | 0 | 10 | 0 |
| DiCalcium Phosphate | 13 | 0 | 13 | 0 |
| Calcium Carbonate | 5.5 | 0 | 5.5 | 0 |
| Potassium Citrate, 1 H2O | 16.5 | 0 | 16.5 | 0 |
|  |  |  |  |  |
| Vitamin Mix V10001 | 10 | 40 | 10 | 40 |
| Choline Bitartrate | 2 | 0 | 2 | 0 |
| **Total** | **1055.05** | **4057** | **649.35** | **2434** |
| **kcal/gm** | **3.85** |  | **3.75** |  |

**Table S3: Primary antibodies used in this study**

| Name | Host | Manufacturer | Catalog number | Dilution |
| --- | --- | --- | --- | --- |
| H3K27ac | rabbit | Abcam, Cambridge, U.K. | ab4729 | 1:100 |
| H3K4me1 | rabbit | Abcam, Cambridge, U.K. | Ab8895 | 1:100 |
| HNF4α | mouse | Abcam, Cambridge, U.K. | ab41898 | 1:100 |

**Table S4: Sequences of primers for RT‐qPCR.**

| Gene | Forward Sequence | Reverse Sequence |
| --- | --- | --- |
| *Ucp1* | AGGCTTCCAGTACCATTAGGT | CTGAGTGAGGCAAAGCTGATTT |
| *Pgc1α(Ppargc1a)* | TATGGAGTGACATAGAGTGTGCT | CCACTTCAATCCACCCAGAAAG |
| *Dio2* | AATTATGCCTCGGAGAAGACCG | GGCAGTTGCCTAGTGAAAGGT |
| *Cidea* | TGACATTCATGGGATTGCAGAC | GGCCAGTTGTGATGACTAAGAC |
| *CD11b* | ATGGACGCTGATGGCAATACC | TCCCCATTCACGTCTCCCA |
| *Ccl2* | TTAAAAACCTGGATCGGAACCAA | GCATTAGCTTCAGATTTACGGGT |
| *Cxcl9* | GGAGTTCGAGGAACCCTAGTG | GGGATTTGTAGTGGATCGTGC |
| *IL-10* | GCTCTTACTGACTGGCATGAG | CGCAGCTCTAGGAGCATGTG |
| *CD68* | TGTCTGATCTTGCTAGGACCG | GAGAGTAACGGCCTTTTTGTGA |
| *CD80* | ACCCCCAACATAACTGAGTCT | TTCCAACCAAGAGAAGCGAGG |
| *F4/80* | TGACTCACCTTGTGGTCCTAA | CTTCCCAGAATCCAGTCTTTCC |
| *CD163* | ATGGGTGGACACAGAATGGTT | CAGGAGCGTTAGTGACAGCAG |
| *CD206* | CTCTGTTCAGCTATTGGACGC | CGGAATTTCTGGGATTCAGCTTC |
| *Cyp7a1* | GGGATTGCTGTGGTAGTGAGC | GGTATGGAATCAACCCGTTGTC |
| *Cyp7b1* | GGAGCCACGACCCTAGATG | TGCCAAGATAAGGAAGCCAAC |
| *Cyp8b1* | CCTCTGGACAAGGGTTTTGTG | GCACCGTGAAGACATCCCC |
| *CYP27a1* | CCAGGCACAGGAGAGTACG | GGGCAAGTGCAGCACATAG |
| *PPARα* | AGAGCCCCATCTGTCCTCTC | ACTGGTAGTCTGCAAAACCAAA |
| *Hnf4α* | CACGCGGAGGTCAAGCTAC | CCCAGAGATGGGAGAGGTGAT |
| *Gapdh* | AGGTCGGTGTGAACGGATTTG | TGTAGACCATGTAGTTGAGGTCA |

**Table S5: Sequences of primers for ChIP‐qPCR.**

| Gene | Forward Sequence | Reverse Sequence |
| --- | --- | --- |
| *Cyp8b1* | CTCCTAGCACTGTACACCAC | GCCTCTGAGCAAAGTCCAAG |
| *Input* | ATGTACCTGCGTCTTCTCCA | CACTGAGTGCTGGGATTACA |
