# Supplementary material for: Caloric restriction remodels the hepatic chromatin landscape and bile acid metabolism by modulating the gut microbiota
Source: Genome Biol. 2023 Apr 30;24:98. doi: 10.1186/s13059-023-02938-5 (PMC10150505; doi:10.1186/s13059-023-02938-5)
Supplement: Supplementary file 8 — Additional file 8. Review history. [file 13059_2023_2938_MOESM8_ESM.docx]

Review History

**First round of review**

**Reviewer 1**

**Are you able to assess all statistics in the manuscript, including the appropriateness of statistical tests used?** Yes, and I have assessed the statistics in my report.

**Comments to author:**

This study illuminates the interaction between gut microbiota, host epigenome, gene expression, and bile acids (BA) synthesis in the modulation of metabolic

adaptation in mice. This is done by analysing the impact of short-term (6 weeks) caloric restriction diet.

The authors show that short-term CR promotes many aspects of metabolic health (Figure 1) and leads to clear changes in the gut microbiome composition, identifying Lactobacillus as prevalent predominant taxum in CR mice (Figure 2). This is linked to changes in the liver metabolic profile, including BAs (Figure 3). The authors show CR altered key chromatin modifications over regulatory elements (Histone H3K27ac, H3K4m1, mostly over bona fide enhancers) and these are linked to the transcriptional regulation of genes involved in metabolic processes (Figure 4). Such changes were linked to various transcriptional regulators by motif analysis, including HNF4alpha and, interestingly, the level of this protein decreased on CR in the liver. ChIP-seq analysis for HNF4alpha shows changes in its binding profile genome-wide, which, in turn, could be linked to changes in chromatin (Figure 5). Importantly, features of improved metabolic health could be transferred through the microbiome from CR mice to high-fat diet (HFD) fed mice via fecal matter transfer and this was linked to changes in the microbiome of the HFD-fed mice (Figure 6). This was linked to changes in hepatic BA profiles and bindings of HNF4alpha to one key gene, Cyb8b1, involved in BA metabolism (Figure 7). Together, the findings support the hypothesis that beneficial improvements of metabolic health via CR are (in part) due to gut microbiota.

The study is interesting for three reasons:

(1) It provides evidence that beneficial effects of even a relatively short term CR are mediated by the microbiome;

(2) It shows that this involves transcription regulator HNF4alpha;

(3) And this is evident in BA generation.

The data are of high quality.

However, some analysis could be worked out more and there could be some improvements as detailed below:

(1) The ChIP-seq data of HNF4alpha could be 'milked' better, more can be extracted from this. What is the overlap between changes in HNF4alpha peaks and gene expression and gene expression changes on CR (if any, Venn diagrams, statistical analysis of overlap, Xi-square etc).

(2) How do the HNF4alpha ChIP-seq data relate to the ATAC-seq data (that did predict a link to HNF4alpha)? This is strangely not explored.

(3) The statistical analyses appear to be not always state-of-the art. For example, in Figures 1 S-U, 3 N-S, 7 D, Supplementary Figures 1 the authors perform simple t-tests over analysis of several features where there should be probably some multiple testing correction or ANOVA tests. For group comparison (e.g., microbiome diversity analyses), we usually perform non-parametric tests, e.g., Kruskal-Wallis followed by two-sided Mann-Whitney post hoc analysis with Holm correction. How were p-values derived for Figure 2B? This should probably be a PERMANOVA test.

Further issues:

Legend Figure 4 G-K: Does this relate all to H3K4me1? This should be made clear in the legend.

Methods section, mice: The mice were treated with an antibiotic cocktail in the drinking water, but the authors also write "We used gastric gavage of oral antibiotics when necessary..", what do the authors mean with "when necessary"? This should be detailed.

Page 6, bottom:

"Real-time PCR confirmed that markers of adipose browning were increased in AL male mice (Figure 1S), whereas pro-inflammatory markers were reduced and anti-inflammatory markers were increased in WAT or liver (Figure 1T-U)."

This statement is confusing, it looks like many inflammation markers are decreased in CR mice.

Page 4, line 85:

"Although epigenetic modifications are relative stable and cell-type specific, these alterations still be flexibly fine-tuned in response to environmental challenges and can impact gene expressions [11]."

Presumably should be

"Although epigenetic modifications are relative stable and cell-type specific, these alterations still need to be flexibly fine-tuned in response to environmental challenges and can impact gene expressions [11]."

Page 6, line 123:

"serum fat-derived hormone leptin", leptin is not 'fat-derived', maybe "fat responsive'?

Page 7, second last line: "OTUs" not "OUTs".

Page 10, line 204: "Gene expression was regulated by regulatory elements, such as enhancers" should presumably be "Gene expression is regulated by regulatory elements, such as enhancers".

Page 17, line 263: "..to the its receptors..", presumably "…to its receptors..".

**Reviewer 2**

**Are you able to assess all statistics in the manuscript, including the appropriateness of statistical tests used?** Yes, and I have assessed the statistics in my report.

**Comments to author:**

In this manuscript, Fan et al have shown that short term CR leads to changed gut microbial diversity remodeled the hepatic enhancer landscape at genomic loci that were enriched for binding sites for signal responsive transcription factors including HNF4a. As a result, liver transcription profile was altered which involve the genes enriched in bile acid metabolism. Furthermore, they show that transferring CR gut microbiota into high fat diet mice recapitulated the features of CR related bile acids metabolism along with attenuated fatty liver. Overall, this is an interesting study, which extensively profiles how gut microbiome, liver epigenome, transcriptome metabolome after short term CR. The author also provided some insight into the interaction between the gut microbiome change and liver epigenome and transcriptome change by transplanting CR gut microbiota to HFD mice. This is a carefully conducted and well-described study. However, there are a lot mistakes should be addressed in the data presentation:

1. There are some conclusions in the text were made with no significant changes in the figure.(e.g Fig 1C liver mass, Fig 1G TG, Fig 6H-L)

2. Fig 1S is not consistent with the description in the text.

3. Please specify the comparison of the volcano plot of the FoldChange like CRvsAL. It is not very clear.

4. In Fig 3B-C, the analysis was based on up or down or both DEG? Please specify it.

5. In Fig 3D-E, what the heatmap showed is opposite to the text. DNL genes increased in CR group from the heatmap in Fig 3D.

6. In Fig3N-S, there are no figures for DCA and TLCA, but mentioned in the text. Please show the plot for these two acids.

7. A peak annotation plot will be helpful to show the H3K27ac CR gain and loss regions are enhancers in Fig 4.

8. Fig 4E is not consistent with the figure legend described.

9. Nomenclature should be consistent. In some figures HNF4a was used, while others Hnf4a was used.

10. How to explain the difference between Fig6Q and Fig2I?

11. In line 311, you refer Fig5D to show the increased binding of Hnf4a near Cyp8b1, but there is no Cyp8b1 information in Fig5D. Please check.

12. Scare bar should be added in the histology figures.

**Authors Response**

**Point-by-point responses to the reviewers’ comments:**

Reviewer reports:

**Reviewer #1: GBIO-D-22-00883**

Calorie Restriction Remodels Hepatic Chromatin Landscape and Bile Acid Metabolism by Modulating the Gut Microbiota

Fan et al.

This study illuminates the interaction between gut microbiota, host epigenome, gene expression, and bile acids (BA) synthesis in the modulation of metabolic adaptation in mice. This is done by analysing the impact of short-term (6 weeks) caloric restriction diet.

The authors show that short-term CR promotes many aspects of metabolic health (Figure 1) and leads to clear changes in the gut microbiome composition, identifying Lactobacillus as prevalent predominant taxum in CR mice (Figure 2). This is linked to changes in the liver metabolic profile, including BAs (Figure 3). The authors show CR altered key chromatin modifications over regulatory elements (Histone H3K27ac, H3K4m1, mostly over bona fide enhancers) and these are linked to the transcriptional regulation of genes involved in metabolic processes (Figure 4). Such changes were linked to various transcriptional regulators by motif analysis, including HNF4alpha and, interestingly, the level of this protein decreased on CR in the liver. ChIP-seq analysis for HNF4alpha shows changes in its binding profile genome-wide, which, in turn, could be linked to changes in chromatin (Figure 5). Importantly, features of improved metabolic health could be transferred through the microbiome from CR mice to high-fat diet (HFD) fed mice via fecal matter transfer and this was linked to changes in the microbiome of the HFD-fed mice (Figure 6). This was linked to changes in hepatic BA profiles and bindings of HNF4alpha to one key gene, Cyb8b1, involved in BA metabolism (Figure 7). Together, the findings support the hypothesis that beneficial improvements of metabolic health via CR are (in part) due to gut microbiota.

The study is interesting for three reasons:

(1)	It provides evidence that beneficial effects of even a relatively short term CR are mediated by the microbiome;

(2)	It shows that this involves transcription regulator HNF4alpha;

(3)	And this is evident in BA generation.

The data are of high quality.

However, some analysis could be worked out more and there could be some improvements as detailed below:

Response: We thank the reviewer for the positive comments, and we revised the manuscript point by point carefully.

(1)	The ChIP-seq data of HNF4alpha could be 'milked' better, more can be extracted from this. What is the overlap between changes in HNF4alpha peaks and gene expression and gene expression changes on CR (if any, Venn diagrams, statistical analysis of overlap, Xi-square etc).

Response: Thanks for your valuable suggestion. We added the overlap analysis between changes in HNF4α peaks and gene expression using HNF4α ChIP-seq and RNA-seq data in **Figure 5**. Among all the DEGs, 440 genes were overlapped with differential HNF4α peaks. The differential HNF4α peaks were significantly correlated with differential gene expressions.

*“We next leveraged our HNF4α ChIP-seq and RNA-seq data to determine whether differential HNF4α binding was accompanied by altered gene expressions. We assigned the differential HNF4α binding peaks to the nearest genes by ChIPseeker and observed around 440 DEGs were overlapped (p-value <  0.0001,* ***Figure 5I****). DEGs with CR-gained HNF4α binding had significantly higher expression (p-value  <  0.0001,* ***Figure. 5J-K****).”*

Page 12, line 259-264 in the revised manuscript (highlighted version)

**Figure 5I-K**

(2)	How do the HNF4alpha ChIP-seq data relate to the ATAC-seq data (that did predict a link to HNF4alpha)? This is strangely not explored.

Response: Thanks for your constructive suggestion. We added further analysis about HNF4α ChIP-seq and ATAC-seq data in **Figure 4** and **Figure 5**. We performed motif analysis in differential open chromatin regions or regions with differential H3K27ac enrichement and found both of them enriched with HNF4α binding motif (**Figure 4**). From heatmap and metagene plot, we observed increased signal of HNF4α binding cites was accompanied by increased ATAC signal, and vice versa. Both regions were enriched for H3K27ac, which indicated that activated HNF4α was localized at activated promoter or enhancer loci (**Figure 5**). All these data indicated potential fuction of HNF4α in chromatin remodeling.

*“Totally, 1074 regions with increased and 4971 regions with decreased chromatin accessibility (FDR <  0.05) were screened. Through HOMER, we also observed enrichment of HNF4α and PPARα binding motifs (****Figure 4J****).*

Page 11, line 228-230 in the revised manuscript (highlighted version)

*“We next performed overlap analysis between the differentially enriched H3K27ac regions and differentially open chromatin regions. Around 673 regions were overlapped and enriched with HNF4α binding sites (****Figure 4K-L****)”*

Page 11, line 230-233 in the revised manuscript (highlighted version)

**Figure 4J-L**

*“To investigate whether CR affect the distribution of HNF4α, we carried out HNF4α ChIP-seq and identified 5935 gain regions and 3841 loss regions (****Figure 5B****). We observed that* *increased signal of HNF4α binding cites was accompanied by increased ATAC signal, and vice versa.* *Both regions were enriched for H3K27ac, which indicated that activated HNF4α was localized at activated promoter or enhancer loci(****Figure 5B-C****).”*

Page 12, line 250-255 in the revised manuscript (highlighted version)

**Figure 5B-C**

(3) The statistical analyses appear to be not always state-of-the art. For example, in Figures 1 S-U, 3 N-S, 7 D, Supplementary Figures 1 the authors perform simple t-tests over analysis of several features where there should be probably some multiple testing correction or ANOVA tests.

Response: Thanks for your valuable advice. We applied multiple testing correction in the reanalysis. The "false discovery rate (FDR)" control the expected proportion of false discoveries amongst the rejected hypotheses. Since there is no conventional cutoff of FDR as the classical significance level of 0.05 for *p*-values, we used 0.1 as an acceptable FDR in the study. After multiple testing correction, the conclusions were the same as before.

** FDR* < 0.1*, ** FDR* < 0.05*, *** FDR* < 0.01 *and **** FDR* < 0.001.

**Figure 1S-U**

**Figure 3N-S**

**Figure 7D-L**

**Supplemental Figure 1**

**Supplemental Figure 4**

For group comparison (e.g., microbiome diversity analyses), we usually perform non-parametric tests, e.g., Kruskal-Wallis followed by two-sided Mann-Whitney post hoc analysis with Holm correction. How were p-values derived for Figure 2B? This should probably be a PERMANOVA test.

Response: Thanks for the comments. We applied Adonis (Permutational multivariate analysis of variance, PERMANOVA) to compare beta diversity of these two groups in **Figure 2B**. PERMANOVA is a non-parametric test of significant difference between two or more groups, based on any distance measure (usually beta diversity). Here we used both bray-curtis and weighted-unifrac distance. Kruskal-Wallis followed by two-sided Mann-Whitney post hoc analysis was often used to compare the alpha diversity between two groups, and we used Kruskal-Wallis test in alpha diversity comparison in our data. The conclusions were in consistent with our previous version.

**Figure 2D-G**

**Further issues:**

Legend Figure 4 G-K: Does this relate all to H3K4me1? This should be made clear in the legend.

Response: Thanks for your suggestion. We have added more details in the figure legends in the revised manuscripts. The Figure 4G, I, M-O were all related to H3K27ac data.

"***(G)*** *Distance to TSS of gain or loss H3K27ac regions between AL and CR groups (n=2 per group).*

***(I)*** *Motifs analysis of differentially H3K27ac enriched regions in AL or CR group (n=2 per group).*

***(M-N)*** *GO pathway analysis of loci near CR gain or loss H3K27ac regions.*

***(O)*** *Gene expression in CR gain or loss H3K27ac regions (n=2 per group).*"

Page 37, line 779-789 in the revised manuscript (highlighted version)

Methods section, mice: The mice were treated with an antibiotic cocktail in the drinking water, but the authors also write "We used gastric gavage of oral antibiotics when necessary..", what do the authors mean with "when necessary"? This should be detailed.

Response: Thanks for the comments. We added more details in the revised manuscripts.

*"Recipient male mice were used gastric gavage of oral antibiotics and the antibiotics were renewed every day."*

Page 19, line 418-419 in the revised manuscript (highlighted version)

Page 6, bottom:

"Real-time PCR confirmed that markers of adipose browning were increased in AL male mice (Figure 1S), whereas pro-inflammatory markers were reduced and anti-inflammatory markers were increased in WAT or liver (Figure 1T-U)."

This statement is confusing, it looks like many inflammation markers are decreased in CR mice.

Response: Thanks for the comments. We revised the text in the manuscript.

*"Real-time PCR confirmed that markers of adipose browning were increased in CR male mice (****Figure 1S****), whereas pro-inflammatory markers were reduced and anti-inflammatory markers were increased in WAT or liver* ***(Figure 1T-U)****."*

Page 6, line 129-132 in the revised manuscript (highlighted version)

Page 4, line 85:

"Although epigenetic modifications are relative stable and cell-type specific, these alterations still be flexibly fine-tuned in response to environmental challenges and can impact gene expressions [11]."

Presumably should be

"Although epigenetic modifications are relative stable and cell-type specific, these alterations still need to be flexibly fine-tuned in response to environmental challenges and can impact gene expressions [11]."

Response: Thanks for the comments. We revised the text according to your suggestion.

*"Although epigenetic modifications are relative stable and cell-type specific, these alterations still need to be flexibly fine-tuned in response to environmental challenges and can impact gene expressions [11]."*

Page 4, line 85-88 in the revised manuscript (highlighted version)

Page 6, line 123:

"serum fat-derived hormone leptin", leptin is not 'fat-derived', maybe "fat responsive'?

Response: Thanks for the comments. We revised the description according to your suggestion in the manuscript.

Page 6, line 124 in the revised manuscript (highlighted version)

Page 7, second last line: "OTUs" not "OUTs".

Response: Thanks for the comments. We revised the text in the manuscripts.

*"Of note, 3 OTUs (OTU3, OTU40 and OTU22) at the genus level of Lactobacillus and 2 OTUs (OTU204 and OTU105) in its species level of reuteri were all significantly enriched after short-term CR."*

Page 7, line 153-156 in the revised manuscript (highlighted version)

Page 10, line 204: "Gene expression was regulated by regulatory elements, such as enhancers" should presumably be "Gene expression is regulated by regulatory elements, such as enhancers".

Response: Thanks for the comments. We corrected the sentence in the revised manuscripts.

*"**Gene expression is regulated by regulatory elements, such as enhancers."*

Page 10, line 206 in the revised manuscript (highlighted version)

Page 17, line 263: "..to the its receptors..", presumably "…to its receptors..".

Response: Thanks for the comments. We corrected the sentence in the revised manuscripts.

*"In the enterohepatic circulation, BAs are endogenous signaling factor that usually bind to its receptors to maintain BA homeostasis …"*

Page 18, line 376-378 in the revised manuscript (highlighted version)

**Reviewer #2: ===**

In this manuscript, Fan et al have shown that short term CR leads to changed gut microbial diversity remodeled the hepatic enhancer landscape at genomic loci that were enriched for binding sites for signal responsive transcription factors including HNF4a. As a result, liver transcription profile was altered which involve the genes enriched in bile acid metabolism. Furthermore, they show that transferring CR gut microbiota into high fat diet mice recapitulated the features of CR related bile acids metabolism along with attenuated fatty liver. Overall, this is an interesting study, which extensively profiles how gut microbiome, liver epigenome, transcriptome metabolome after short term CR. The author also provided some insight into the interaction between the gut microbiome change and liver epigenome and transcriptome change by transplanting CR gut microbiota to HFD mice. This is a carefully conducted and well-described study. However, there are a lot mistakes should be addressed in the data presentation:

Response: We thank the reviewer for the constructive comments and we carefully revised the manuscript point by point.

1.	There are some conclusions in the text were made with no significant changes in the figure.(e.g Fig 1C liver mass, Fig 1G TG, Fig 6H-L)

Response: Thanks for the comments. We revised the text in the conclusion according to your comments.

*"BAT mass and WAT mass (epididymal and inguinal WAT) significantly decreased in CR group, while liver mass decreased with no significant difference (****Figure 1C****)."*

Page 6, line 114-116 in the revised manuscript (highlighted version)

*"CR showed improved glucose-insulin homeostasis (****Figure 1E-H****) along with lower serum levels of glucose* ***(Figure 1I****) and total cholesterol (TC), low-density lipoprotein (LDL) (****Figure 1J****)."*

Page 6, line 118-120 in the revised manuscript (highlighted version)

*"We also performed GTT and ITT test and the ITT test results indicated that receiving microbiota from CR mice could improve insulin sensitivity in obese mice (****Figure 6H-L****)."*

Page 13, line 280-283 in the revised manuscript (highlighted version)

2.	Fig 1S is not consistent with the description in the text.

Response: Thanks for the comments. We revised the text in the manuscript.

*"Real-time PCR confirmed that markers of adipose browning were increased in CR male mice (****Figure 1S****)"*

Page 6, line 129-130 in the revised manuscript (highlighted version)

3.	Please specify the comparison of the volcano plot of the FoldChange like CRvsAL. It is not very clear.

Response: Thanks for the comments. We added the FoldChange in figure legend in the revised manuscript.

*"****(A)*** *Volcano plots of differentially expressed genes (|Fold Change| >2) between AL and CR groups (n=4 per group)."*

Page 35, line 749-750 in the revised manuscript (highlighted version)

*"****(H)*** *Volcano plots of differential metabolites (|Fold Change| >2) between AL and CR groups (n=9-10 per group)."*

Page 36, line 755-757 in the revised manuscript (highlighted version)

*"****(K)*** *Volcano plots of differential bile acids (|Fold Change| >2) between AL and CR groups (n=9-10 per group)."*

Page 36, line 759-761 in the revised manuscript (highlighted version)

4.	In Fig 3B-C, the analysis was based on up or down or both DEG? Please specify it.

Response: Thanks for the comments. The GO and KEGG analysis were based on both up and down DEGs induced by AL or CR. We added the description in the revised manuscript.

*"****(B-C)*** *GO and KEGG pathway analysis of differentially expressed genes (both up and down) induced by AL or CR (n=4 per group)."*

Page 35, line 750-752 in the revised manuscript (highlighted version)

5.	In Fig 3D-E, what the heatmap showed is opposite to the text. DNL genes increased in CR group from the heatmap in Fig 3D.

Response: Thanks for the comments. We corrected it in the revised manuscript.

*"In CR group, lipid metabolism was altered characterized by upregulation of genes mediating DNL (****Figure 3D****) and downregulation of genes in FAO (****Figure 3E****)."*

Page 8, line 173-175 in the revised manuscript (highlighted version)

6.	In Fig3N-S, there are no figures for DCA and TLCA, but mentioned in the text. Please show the plot for these two acids.

Response: Thanks for your comments. We showed the plots of DCA and TLCA in the **Supplemental Figure 1A** and **Supplemental Figure 1F**. We added the description in the revised manuscript.

*"Of these, quantitative analysis indicated that the conjugated BAs glycocholic acid (GCA), taurolithocholic acid (TLCA), taurodeoxycholic acid (TDCA), taurocholic acid (TCA) were significantly elevated in the CR mice. The levels of unconjugated BAs such as hyodeoxycholic acid (HDCA), ursocholic acid (UCA) and beta-Muricholic acid (beta-MCA) were significantly decreased while other bile acids levels were not changed in the CR group (****FDR < 0.1,*** ***Figure 3N-S****,* ***Supplemental Figure 1****)."*

Page 9, line 193-199 in the revised manuscript (highlighted version)

**Supplemental Figure 1**

7.	A peak annotation plot will be helpful to show the H3K27ac CR gain and loss regions are enhancers in Fig 4.

Response: Thanks for your suggestion. We added peak annotation in **Figure 4.**

*“To annotate the location of H3K27ac CR gain and loss peaks in terms of genomic features, we used ChIPseeker to assign peaks to genomic annotation in the Distal Intergenic, Exon, Intron, Promoter and UTR. We found that over 25% peaks were far from the gene promoters in CR gain and loss regions (****Figure 4H****), which might be enhancers.”*

Page 10, line 217-221 in the revised manuscript (highlighted version)

**Figure 4H**

8.	Fig 4E is not consistent with the figure legend described.

Response: Thanks for your comments. We modified the figure legend in the revised manuscript.

*“****(E-F)*** *Differentially representative enriched region of open chromatin, H3K27ac, H3K4me1 and gene expression levels in AL and CR groups (n=2 per group).* *(E) depicts the Cyp7b1 locus which has lower chromatin accessbility, acetylation, and transcript levels in CR group. (F) depicts the Fmo3 locus which has higher acetylation, methylation and transcript levels in CR group.”*

Page 37, line 775-779 in the revised manuscript (highlighted version)

9.	Nomenclature should be consistent. In some figures HNF4a was used, while others Hnf4a was used.

Response: Thanks for the comments. We uniformed the nomenclature of HNF4α in the revised manuscript. For protein and transcription factor, we used uppercase HNF4α; while for gene, we used lowercase italic *Hnf4α*.

10.	How to explain the difference between Fig6Q and Fig2I?

Response: Thanks for the question. The model in **Figure 6** and **Figure2** were different. The mice in **Figure 2** were on the control diet, while the mice in **Figure 6** were on the high fat diet (HFD). Actually, microbiota transplation was a “drity” experiment. During the process, the oxygen, the preparation method and transplantion procedure would casue the shiftness of the bacteria composition. After the transplation, the microbiota could be also changed by the HFD. Here, we used random forests method to construct a classification model to screen the functional OTUs in CR model or in fecal microbiota transplantation model. Notably, microbiota-depleted mice fed the high fat diet (HFD, 60% kcal) were transplanted with CR microbiota (HFDR) resembled partly the phylum-level proportional abundance of donor mice following CR (**Figure 2H**, **Figure 6O**). That maybe partialy explained the differecen observed in **Fig6Q** and **Fig2I**. Since we had several candidate bacteria now, we will perform a more “clean” experiment using single strain bacteria in the transplantation in the next study.

11.	In line 311, you refer Fig5D to show the increased binding of Hnf4a near Cyp8b1, but there is no Cyp8b1 information in Fig5D. Please check.

Response: Thanks for your comments. In the **Figure 5F**, we now showed HNF4α and H3K27ac enrichement regions near *Cyp8b1* of between AL and CR groups (n=2 per group). We added the description of *Cyp8b1* in figure legend.

*"****(F)*** *Representative examples Cyp8b1 of HNF4α and H3K27ac enriched regions between AL and CR groups (n=2 per group)."*

Page 38, line 800-801 in the revised manuscript (highlighted version)

12.	Scare bar should be added in the histology figures.

Response: Thanks for your suggestion. We added scare bar (×20 magnification; scale bar indicates 100 μm) in **Figure 1D** and **Figure 6D**.

**Figure 1D**

**Figure 6D**

**Second round of review**

**Reviewer 1**

The authors addressed the reviewers' comments and concerns carefully and this interesting paper should now be accepted.

I found a few minor issues that the authors should address and correct, listed below. I do not think it is necessary to re-review after these corrections have been implemented:

Figure 4K (Venn diagram):

Can the authors please state the methods of analysis, is this comparison by gene (peaks assigned to genes etc, like in Fig. 5I)? I would be good if they state some level of significance for the overlap, such as X-square test.

Legend Figure 5D:

“(D) Representative examples Fom3 of HNF4α and H3K27ac..” this sentence dos not make sense, please re-write. This might be better: “ChIP-seq of HNF4α and H3K27ac at the Fom3 locus as a representative example” (or similar).

Page 12, line 252: ‘cites’ should be ‘sites’

**Reviewer 2**

The author answered all my questions and incorporated my comment. This is a improved and interesting study that now warrants publication in Genome Biology.

**Authors Response**

**Point-by-point responses to the reviewers’ comments:**

Reviewer #1: The authors addressed the reviewers' comments and concerns carefully and this interesting paper should now be accepted.

I found a few minor issues that the authors should address and correct, listed below. I do not think it is necessary to re-review after these corrections have been implemented:

Response: We are glad that the reviewer 1 is satisfied with our revised manuscript. Thanks again for the constructive comments.

Figure 4K (Venn diagram):

Can the authors please state the methods of analysis, is this comparison by gene (peaks assigned to genes etc, like in Fig. 5I)? I would be good if they state some level of significance for the overlap, such as X-square test.

Response: Thanks for the comment. We did overlap analysis using Chi-squared test as reviewer 1 suggested in Figure 4K and the *p* value was < 2.2e-16. We added this in the methods now.

*“The statistical significance of the overlap was determined by Chi-squared test.”*

ATAC peak 51897

H3K27ac peak 40576

Total peak 91800

Legend Figure 5D:

"(D) Representative examples Fom3 of HNF4α and H3K27ac.." this sentence dos not make sense, please re-write. This might be better: "ChIP-seq of HNF4α and H3K27ac at the Fom3 locus as a representative example" (or similar).

Response: Thanks for the comments, we revised the figure legends in the highlighted manuscripts.

*“****(D)*** *ChIP-seq of HNF4α and H3K27ac at the Fom3 locus as a representative example (n=2 per group).* ***(E)*** *ChIP-seq of HNF4α and H3K27ac at the Cyp7b1 locus as a representative example (n=2 per group).* ***(F)*** *ChIP-seq of HNF4α and H3K27ac at the Cyp8b1 locus as a representative example (n=2 per group).”*

Page 12, line 252: 'cites' should be 'sites'

Response: Done.

*“We observed that increased signal of HNF4α binding sites was accompanied by increased ATAC signal, and vice versa.”*

Reviewer #2:

The author answered all my questions and incorporated my comment. This is a improved and interesting study that now warrants publication in Genome Biology.

Response: We are glad this reviewer is satisfied with our revision. Thanks again for the constructive comments.
